# Airway occlusions to measure inspiratory effort, respiratory drive, and lung mechanics during noninvasive ventilation

**DOI:** 10.1093/ajrccm/aamag293

**Published:** 2026-06-13

**Authors:** Francesco Murgolo, Domenico Luca Grieco, Rossana Soloperto, Michele Bertoni, Savino Spadaro, Nicola Bartolomeo, Tommaso Pettenuzzo, François Beloncle, Delphine Venkatapen, Alessandro Cardu, Tommaso Rosà, Chiara Iacovelli, Paolo Navalesi, Salvatore M Maggiore, Ewan C Goligher, V Marco Ranieri, Lorenzo del Sorbo, Massimo Antonelli, Salvatore Grasso

**Affiliations:** Department of Precision-Regenerative Medicine and Jonic Area, Section of Anesthesiology and Intensive Care Medicine, University of Bari “Aldo Moro,” Bari, Italy; Department of Anesthesia and Critical Care, Policlinico di Bari, Bari, Italy; Interdepartmental Division of Critical Care Medicine, Medical and Surgical Intensive Care Unit, University Health Network, Toronto General Research Institute, Toronto, ON, Canada; Department of Anesthesia, Intensive Care and Emergency, Fondazione Policlinico A. Gemelli IRCCS, Rome, Italy; Department of Anesthesiology and Intensive Care Medicine, Catholic University of The Sacred Heart, Rome, Italy; Department of Precision-Regenerative Medicine and Jonic Area, Section of Anesthesiology and Intensive Care Medicine, University of Bari “Aldo Moro,” Bari, Italy; Department of Anesthesia and Critical Care, Policlinico di Bari, Bari, Italy; Department of Medical and Surgical Specialties, Radiological Sciences and Public Health, University of Brescia, Brescia, Italy; Department of Emergency, ASST Spedali Civili University Hospital, Brescia, Italy; Department of Translational Medicine, Section of Anesthesiology and Intensive Care Medicine, University of Ferrara, Ferrara, Italy; Interdisciplinary Department of Medicine, Section of Statistic, University of Bari Aldo Moro, Bari, Italy; Department of Medicine, University of Padua, Padua, Italy; Vent’Lab, Medical Intensive Care Unit, Angers University Hospital, Angers, France; Medical Intensive Care Unit, Angers University Hospital, Angers, France; Vent’Lab, Medical Intensive Care Unit, Angers University Hospital, Angers, France; Med2Lab Laboratory, Air Liquide Medical Systems, Antony, France; Department of Anesthesia, Intensive Care and Emergency, Fondazione Policlinico A. Gemelli IRCCS, Rome, Italy; Department of Anesthesiology and Intensive Care Medicine, Catholic University of The Sacred Heart, Rome, Italy; Department of Anesthesia, Intensive Care and Emergency, Fondazione Policlinico A. Gemelli IRCCS, Rome, Italy; Department of Anesthesiology and Intensive Care Medicine, Catholic University of The Sacred Heart, Rome, Italy; Department of Anesthesia, Intensive Care and Emergency, Fondazione Policlinico A. Gemelli IRCCS, Rome, Italy; Department of Anesthesiology and Intensive Care Medicine, Catholic University of The Sacred Heart, Rome, Italy; Department of Medicine, University of Padua, Padua, Italy; Institute of Anesthesia and Intensive Care, University Hospital of Padua, Padua, Italy; Department of Anesthesiology and Intensive Care Medicine, Ecclesiastical Entity—Regional General Hospital “F. Miulli,” Bari, Italy; University Department of Medicine and Surgery, Libera Università Mediterranea, Bari, Italy; Interdepartmental Division of Critical Care Medicine, Medical and Surgical Intensive Care Unit, University Health Network, Toronto General Research Institute, Toronto, ON, Canada; Department of Precision-Regenerative Medicine and Jonic Area, Section of Anesthesiology and Intensive Care Medicine, University of Bari “Aldo Moro,” Bari, Italy; Department of Anesthesia and Critical Care, Policlinico di Bari, Bari, Italy; Interdepartmental Division of Critical Care Medicine, Medical and Surgical Intensive Care Unit, University Health Network, Toronto General Research Institute, Toronto, ON, Canada; Department of Anesthesia, Intensive Care and Emergency, Fondazione Policlinico A. Gemelli IRCCS, Rome, Italy; Department of Anesthesiology and Intensive Care Medicine, Catholic University of The Sacred Heart, Rome, Italy; Department of Precision-Regenerative Medicine and Jonic Area, Section of Anesthesiology and Intensive Care Medicine, University of Bari “Aldo Moro,” Bari, Italy; Department of Anesthesia and Critical Care, Policlinico di Bari, Bari, Italy

**Keywords:** noninvasive ventilation, occlusion maneuver, Pocc, inspiratory effort, reintubation

## Abstract

**Rationale:**

In intubated patients, occlusion maneuvers allow noninvasive assessment of inspiratory effort, respiratory drive, and lung mechanics.

**Objectives:**

To assess the feasibility of occlusion maneuvers during noninvasive ventilation (NIV).

**Methods:**

In this multicenter study, 60 hypoxemic patients underwent 2 randomized 1-hour NIV sessions with oronasal and full-face masks after extubation. End-expiratory and end-inspiratory occlusions measured expiratory occlusion pressure (Pocc), 100-ms airway pressure drop (P0.1), and plateau pressure. Esophageal manometry, calibrated before extubation, provided reference values for inspiratory effort, assessed as esophageal pressure swing (ΔPes) and dynamic transpulmonary driving pressure (ΔPL,dyn = pressure support  − ΔPes). Interface-specific conversion factors (K) translating Pocc into predicted ΔPes (K × Pocc) and predicted ΔPL,dyn (pressure support − predicted ΔPes) were derived through 100-interaction cross-validation (20-patient derivation set, 40-patient validation set).

**Measurements and Main Results:**

Pocc was measurable in all patients. Mean K was 0.71 with the oronasal mask and 0.80 with the full-face mask. Predicted ΔPes agreed with observed ΔPes (oronasal: bias −0.41 cm H_2_O, 95% limits of agreement −2.3 to 1.5; full-face: 0.09, −2.9 to 3.1), and predicted ΔPL,dyn agreed with observed ΔPL,dyn (oronasal bias: 0.03, −2.9 to 2.9; full-face: −0.04, −4.3 to 4.2). Predicted ΔPes identified observed ΔPes ≤ −10 cm H_2_O, with areas under the receiver operating characteristic curve of 0.98 (oronasal) and 0.97 (full-face). Ventilator-derived P0.1 did not precisely quantify respiratory drive, but values >2.7 cm H_2_O with the oronasal mask and >3 cm H_2_O with the full-face mask identified high drive with specificity >90%. Plateau pressure was unstable in 78% (oronasal) and 90% (full-face) of patients. More negative predicted ΔPes, higher predicted ΔPL,dyn, and lower predicted lung compliance (expiratory tidal volume/predicted ΔPL,dyn) were associated with subsequent reintubation.

**Conclusions:**

During NIV, Pocc-derived parameters provide noninvasive estimates of inspiratory effort, lung stress, and mechanics, whereas ventilator P0.1 and plateau pressure are less reliable.

At a Glance Commentary **Scientific Knowledge on the Subject:** During invasive assisted ventilation, brief occlusion maneuvers allow measurement of the maximal negative deflection in airway pressure (Pocc) , airway occlusion pressure 0.1 second after the onset of inspiratation (P0.1), and plateau airway pressure, providing bedside quantification of inspiratory effort, respiratory drive, and lung mechanics. However, no clinical studies have validated the feasibility of occlusion maneuvers in patients receiving noninvasive ventilation (NIV).

**What This Study Adds to the Field:** We show that Pocc is feasible during face mask NIV and provides clinically acceptable noninvasive estimates of inspiratory effort, dynamic transpulmonary driving pressure, and lung compliance. Ventilator-derived P0.1 discriminates patients with high respiratory drive, whereas plateau pressure is unreliable.

## Introduction

In hypoxemic patients, noninvasive ventilation (NIV) can improve oxygenation, reduce the work of breathing, and may avert endotracheal intubation.^1^ However, prolonged NIV with delayed intubation may worsen outcomes, particularly in patients with vigorous inspiratory effort.^2-4^ Excessive inspiratory effort may lead to high tidal volumes and transpulmonary driving pressure, thereby increasing global and regional lung stress and contributing to self-inflicted lung injury.^5^

Esophageal manometry is the gold standard for measuring inspiratory effort and transpulmonary driving pressure.^6-8^ Physiological studies have demonstrated the value of monitoring inspiratory effort through esophageal manometry for early identification of patients at risk of NIV failure.^9-11^ However, this technique is invasive and requires dedicated equipment and expertise, limiting its routine use in clinical practice.

To overcome these limitations, noninvasive surrogates have been proposed in intubated patients. An end-expiratory occlusion allows measurement of the negative deflection in airway pressure during the first breath (Pocc), which provides a reliable estimate of inspiratory effort.^12^ The airway pressure drop 100 ms after the beginning of an occluded breath (P0.1) reflects respiratory drive.^13^ A brief end-inspiratory hold allows measurement of plateau pressure (Pplat) and calculation of airway driving pressure.^14^ Whether these metrics are applicable during NIV has not been established yet.

We conducted a multicenter study to assess whether occlusion-derived parameters accurately reflect inspiratory effort, respiratory drive, lung stress, and compliance during NIV. Some of the results of these studies have been previously reported in the form of an abstract.^15^

## Methods

Additional details are provided in the Online Supplement.

### Study design

This multicenter physiological study was conducted in 5 general ICUs in Italy between November 2023 and September 2025. Ethical approval was obtained in each center (coordinating center: Bari University Hospital, approval number 7748-0049191; ClinicalTrials.gov: NCT06344234). Written informed consent was obtained from all enrolled patients according to committee recommendations.

### Study population

Adult intubated patients who had received invasive mechanical ventilation for ≥48 hours and were scheduled for extubation were screened for inclusion. Patients were screened before extubation to allow for correct positioning and calibration of the esophageal catheter, which can only be performed during invasive ventilation.^16^ Hypoxemic, nonhypercapnic (PaO_2_/FiO_2_ ratio <300 mm Hg and PaCO_2_ ≤45 mm Hg) patients with at least one high-risk factor for reintubation were included.^17^^,^^18^ Exclusion criteria were failure of the spontaneous breathing trial,^19^^,^^20^ neuromuscular disease, pregnancy, tracheostomy, acute exacerbation of chronic respiratory disease, refusal of consent, and an air leak >30% during NIV before study initiation.

### Study procedure

After a successful spontaneous breathing trial and the decision to extubate, patients were reconnected to mechanical ventilation for 1 hour.^21^ During this period, an esophageal balloon catheter (Nutrivent, Sidam, Mirandola, Italy) was inserted if not already in place, and esophageal pressure (Pes) was verified using a dynamic occlusion test.^16^ NIV was immediately initiated after extubation using a gas-compressed mechanical ventilator connected through a dual-limb circuit and a heat-and-moisture exchanger. For study purposes, NIV-specific ventilator software was not activated and leak compensation was disabled; NIV was initially set with positive end-expiratory pressure (PEEP) of 5 to 7 cm H_2_O and pressure support of 5 cm H_2_O, then titrated to achieve a tidal volume of 6 to 8 mL/kg predicted body weight; FiO_2_ was adjusted to maintain SpO_2_ between 92% and 96%. Pressurization time was set at 50 to 100 ms and expiratory trigger sensitivity at 20% to 50% of peak inspiratory flow. All patients underwent 2 one-hour NIV sessions in randomized order (computer-generated random sequence), using 2 different interfaces (oronasal and full-face masks; HMC GROUP, Mirandola, Italy). During NIV, 3 nonconsecutive end-expiratory and end-inspiratory occlusion maneuvers were performed to measure Pocc and plateau pressure. P0.1 was measured by the ventilator. Between NIV sessions, high-flow nasal oxygen was administered for 1 hour. After completion of the protocol, patients were managed with alternating high-flow nasal oxygen and NIV, for at least 48 hours, and then according to clinical practice.^22^ The decision to reintubate the patients in case of postextubation distress was made by attending physicians who were blinded to study measurements.

### Measurements

Patients’ demographics and most relevant clinical characteristics were collected at study entry. During study procedures Pocc, P0.1, and Pplat were collected from the ventilator screen; ventilator-derived Pplat was measured after approximately 1 s of inspiratory hold. For offline analysis, airway flow (Fleisch pneumotachograph No. 2, Metabo, Lausanne, Switzerland) was measured at airway opening (Y-piece) together with airway pressure (Paw). Pes and Paw were recorded continuously at 200 Hz and stored for offline analysis (ICU Lab, Kleistek, Bari, Italy). For each patient, 155 to 222 nonoccluded breaths and 3 end-expiratory and 3 end-inspiratory occlusions were analyzed. Tidal volume was obtained as digital integration of flow signal. When measured offline from data recorded by the pneumotachograph, Pocc and P0.1 were defined as the maximal negative Paw deflection and the Paw drop during the first 100 ms, respectively,^23^ whereas Pplat was measured at 0.3 and 1.0 s after the onset of end-inspiratory occlusion (Pplat, 0.3 s and Pplat, 1 s) and its stability was defined as a difference ≤ ±1 cm H_2_O between these 2 values^24^ (Figure 1). Airway driving pressure was Pplat—PEEP. Inspiratory effort was calculated as the negative Pes swing during nonoccluded breaths (actual ΔPes),^25^ averaged over 5 min of stable breathing before occlusions. Respiratory drive was referred as Pes drop at 100 ms during end-expiratory occlusion (P0.1,es).^26^^,^^27^ As a complementary analysis, respiratory drive was also assessed by the maximal rate of esophageal pressure decrease during inspiration (ΔPes/dt) measured on nonoccluded breaths. Transpulmonary pressure was Paw-Pes^16^; dynamic transpulmonary driving pressure (ΔPL,dyn) was the positive swing in transpulmonary pressure from the onset of inspiration to its peak value^12^^,^^14^; dynamic lung compliance (Clung,dyn) was the ratio between expiratory tidal volume and ΔPL,dyn.^26^ For all calculations, the beginning of inspiration was identified at the point of initial Pes decay.^28^ Gas exchange and hemodynamics were recorded at the end of each NIV session.

**Figure 1:**
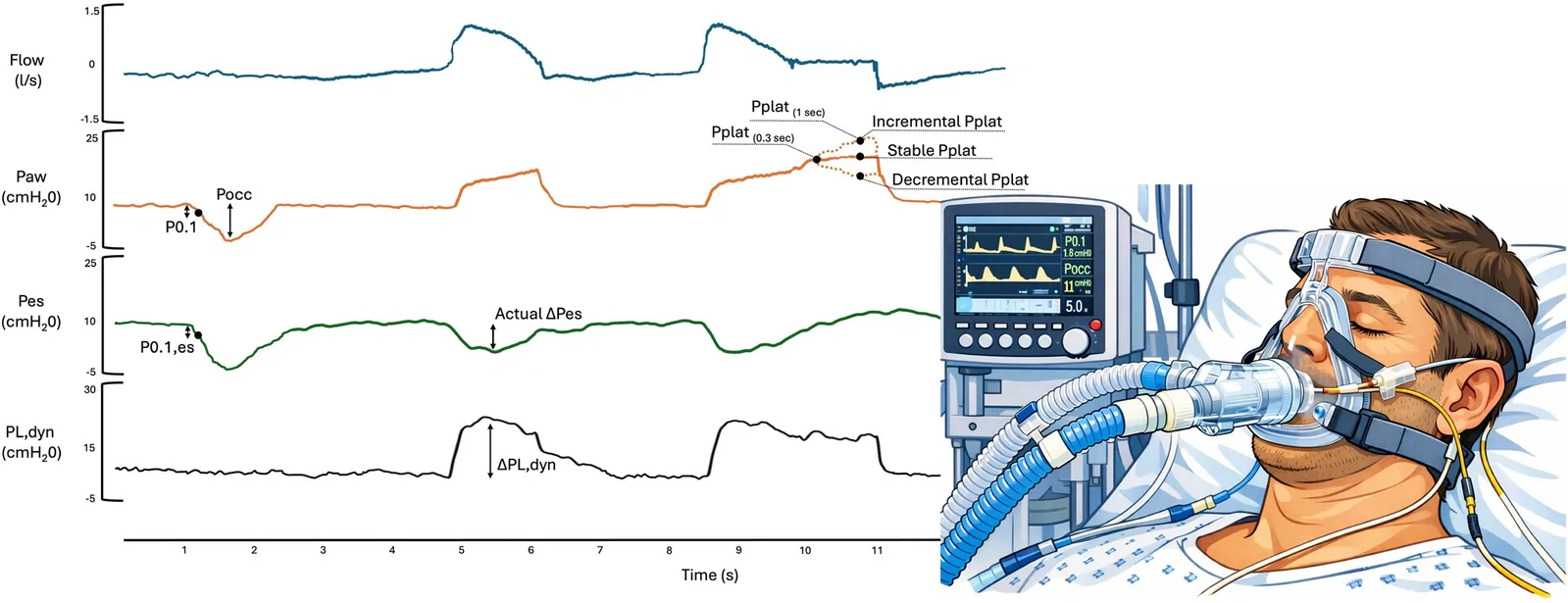
Representative experimental records of airway flow, airway pressure, esophageal pressure, and transpulmonary pressure during end-expiratory and end-inspiratory occlusion maneuvers. Abbreviations: Paw, airway pressure; Pes, esophageal pressure; PL,dyn, dynamic transpulmonary driving pressure; P0.1, airway occlusion pressure 0.1 second after the onset of inspiratory effort; P0.1,es, esophageal pressure deflection 0.1 second after the onset of inspiratory effort; Pocc, maximal negative deflection in airway pressure during an end-expiratory occlusion; Pplat, inspiratory plateau pressure.

### Objective

The primary objective was to assess the feasibility of occlusion maneuvers, the accuracy of derived metrics in estimating inspiratory effort, respiratory drive, lung stress, and mechanics during NIV. The impact of air leaks on these measurements was also assessed. As an exploratory endpoint, we evaluated whether occlusion-derived indices were associated with reintubation within 7 days from extubation.

### Statistical analysis

Data are reported as median (IQR) or number (%). Normality was assessed using the Shapiro–Wilk test. Intrapatient variability was quantified by the coefficient of variation (CV%) and intraclass correlation coefficient (Table S1). Between-group and within-group comparisons used the χ^2^ or Mann–Whitney U tests, and the Wilcoxon signed-rank test, as appropriate. To derive inspiratory effort and lung stress from Pocc, we performed 100 repeated internal cross-validations: At each iteration, patients were randomly assigned to a derivation set (one-third) and a validation set (two-thirds). In the derivation set, we calculated K = actual ΔPes/Pocc and applied it in the validation set to compute predicted ΔPes = K × Pocc and predicted ΔPL,dyn = [Pressure support − (K × Pocc)].^12^ Ventilator-derived Pocc was used to compute predicted ΔPes, and predicted ΔPL,dyn. Associations between measurements were evaluated using mixed-effects models to account for repeated measures, with patients included as random effects and time included as a fixed effect, while agreement was assessed using the concordance correlation coefficient (CCC) and Bland–Altman analysis, both adapted for repeated measurements.^29^^,^^30^ Excessive inspiratory effort and high drive were defined as actual ΔPes ≤ −10 cm H_2_O^9-11^ and P0.1,es >3 cm H_2_O,^27^ respectively. Discriminatory power of predicted ΔPes (for excessive effort) and P0.1 (for high drive), was evaluated by receiver operating characteristic curve analysis with bootstrapped 95% CIs. Extubation failure risk was analyzed by univariable logistic regression using patient-level mean values (averaged across 3 measurements) stratified by NIV interface. Kaplan–Meier analysis for time to reintubation was performed by stratifying patients according to the median value of predicted ΔPes, PL,dyn, and Clung,dyn observed in the extubation failure group; curves were compared using log-rank test. Sample size was determined a priori, yielding a planned total of 60 patients.

**Table 1:** Demographics and baseline characteristics of enrolled patients.

Characteristic	**All patients (*N*** = **60)**
**Demographic characteristics**	
** Age, years**	64 [57-72]
** Male sex, No. (%)**	43 (71.7)
**APACHE II score^a^**	
** At ICU admission**	17 [13-20]
** On day of extubation**	12 [9-16]
**Length of MV before extubation, days**	5 [4-6]
**BMI^b^**	27 [26-29]
**RASS**	1 [0-2]
**Predicted body weight, kg^c^**	61 [54-69]
**Diagnosis at admission, No. (%)**	
** Primary respiratory failure**	37 (61.7)
** Medical**	12 (20.0)
** Postoperative care**	11 (18.3)
**Comorbidities, No. (%)**	
** Arterial hypertension**	18 (30.0)
** Neurologic disease**	12 (20.0)
** Diabetes mellitus**	17 (28.3)
** Renal disease**	11 (18.3)
** Liver disease**	9 (15.0)
** Obesity (BMI >30 kg/m^2^)**	5 (8)
**Baseline ventilation, oxygenation, and hemodynamics before extubation**	
** PEEP, cm H_2_O**	7 [6-8]
** PS, cm H_2_O**	7 [6-8]
** V_T_/PBW**	7.15 [6.90-7.50]
** Respiratory rate, bpm**	21 [18-24]
** Arterial pH**	7.40 [7.38-7.42]
** PaO_2_/FiO_2_ mm Hg**	183 [167-222]
** PaCO_2_, mm Hg**	41 [37-42]
** Systolic arterial pressure, mm Hg**	122 [110-132]
** Diastolic arterial pressure, mm Hg**	76 [68-82]
** Heart rate, bpm**	80 [71-87]

Results are expressed as median [IQR] for continuous variables and number (%) for categorical variables.

Abbreviations: APACHE II, Acute Physiology and Chronic Health Evaluation II; BMI, body mass index; MV, mechanical ventilation; PBW, predicted body weight; PEEP, positive-end expiratory pressure; PS, pressure support delivered by ventilator; RASS, Richmond Agitation and Sedation Scale; V_T_, tidal volume.

a APACHE II score was calculated from 17 variables. Scores range from 0 to 71 points, with higher scores indicating more severe disease.

b Calculated as weight in kilograms divided by height in meters squared.

c Calculated as: 50 [or 45.5 for women] + 0.91× [height [cm] − 152.4].

## Results

Among 106 screened patients, 46 were excluded per predefined criteria (Figure S1). Demographics and most relevant clinical characteristics of the 60 included patients are reported in Table 1. Breathing pattern and clinical variables during oronasal and full-face NIV are summarized in Table 2.

**Table 2:** Breathing pattern and clinical variables during oronasal and full-face NIV.

Characteristic	NIV interface		*P* value
	Oronasal	Full-face	
**Nonoccluded breaths**			
** PEEP, cm H_2_O**	5 [5-6]	5 [5-6]	.83
** Ppeak, cm H_2_O**	11 [11-12]	11 [11-12]	.71
** V_T_/PBW, mL/kg**	7.8 [7.1-9.5]	8.7 [8.1-9.4]	.13
** Respiratory rate, breaths/min**	15 [12-20]	15 [12-21]	.91
** Air leaks (%)**	20 [17-21]	21 [17-23]	.71
** Actual ΔPes, cm H_2_O**	−6 [−10 to −4]	−7 [−11 to −5]	.48
** ΔPL, dyn, cm H_2_O**	12 [10-16]	12 [10-17]	.68
** Clung, dyn, mL/cm H_2_O**	35 [28-45]	32 [27-48]	.24
**Occlusion-derived parameters**			
** Pocc, cm H_2_O**	−9 [−14 to −7]	−9 [−14 to −7]	.65
** Pocc, pneumotach, cm H_2_O**	−9 [−14 to −7]	−10 [−15 to −7]	.98
** P0.1, es, cm H_2_O**	0.8 [0.5-1.2]	0.8 [0.5-1.4]	.72
** P0.1, vent, cm H_2_O**	2.1 [1.9-2.2]	2.6 [2.5-2.8]	.08
** P0.1, pneumotach, cm H_2_O**	2.5 [1.6-3.3]	2.6 [1.5-3.4]	.99
** Pplat, vent, cm H_2_O**	12 [10-14]	12 [10-14]	.72
** Pplat, 0.3 s, cm H_2_O**	11 [11-12]	11 [10-12]	.07
** Pplat, 1 s, cm H_2_O**	12 [9-14]	12 [10-14]	.60
**Gas exchange and hemodynamics collected at the end of NIV session**			
** Arterial pH**	7.41 [7.38-7.44]	7.41 [7.38-7.44]	.08
** PaO_2_/FiO_2_ mm Hg**	212 [184-260]	223 [188-255]	.46
** PaCO_2_, mm Hg**	42 [37-44]	43 [38-44]	.35
** Systolic arterial pressure, mm Hg**	123 [112-135]	125 [112-139]	.15
** Diastolic arterial pressure, mm Hg**	80 [75-86]	81 [73-86]	.55
** Heart rate, bpm**	76 [66-82]	73 [68-84]	.55

Results are expressed as median [IQR].

Abbreviation: ΔPL,dyn, dynamic transpulmonary driving pressure; Actual ΔPes, maximal swing in esophageal pressure recorded during nonoccluded breath (reference metric for inspiratory effort); Clung,dyn, dynamic lung compliance; NIV, noninvasive ventilation; P0.1, airway occlusion pressure at 100 ms; P0.1,es, esophageal pressure drop 100 ms after the onset of inspiratory effort (reference metric for respiratory drive); PEEP, positive end-expiratory pressure; Pes, esophageal pressure; pneumotach, pneumotachograph-derived; Pocc, airway occlusion pressure; Ppeak, Peak airway opening pressure; Pplat, inspiratory plateau pressure recorded at 0.3 s (Pplat, 0.3 sec) and 1.0 s (Pplat, 1 sec) from the start of the end-inspiratory hold; vent, ventilator-derived; V_T_/PBW, inspired tidal volume normalized to predicted body weight.

### Pocc and inspiratory effort

Pocc was available in all patients with both interfaces (Figure S2). In the derivation cohort, the conversion factor K (actual ΔPes/Pocc) was 0.71 (95% CI, 0.68-0.75) for oronasal and 0.80 (95% CI, 0.79-0.81) for full-face NIV. In the validation cohort, predicted ΔPes (K × Pocc) showed good agreement with actual ΔPes (oronasal: CCC, 0.92 [95% CI, 0.90-0.94], bias −0.41 cm H_2_O, limits −2.3 to 1.5; full-face: CCC, 0.93 [95% CI, 0.91-0.94], bias 0.09 cm H_2_O, limits −2.9 to 3.1) (Figure 2A); visual inspection of the Bland–Altman plots suggested heteroscedasticity, with lower precision at higher levels of inspiratory effort (Table S3). Actual ΔPes was ≤ −10 cm H_2_O in 19 patients during oronasal and 21 during full-face NIV; predicted ΔPes < −9 cm H_2_O accurately identified excessive effort (area under the curve, 0.98 [95% CI, 0.97-0.99] and 0.97 [95% CI, 0.96-0.99], respectively) (Table S2).

**Figure 2:**
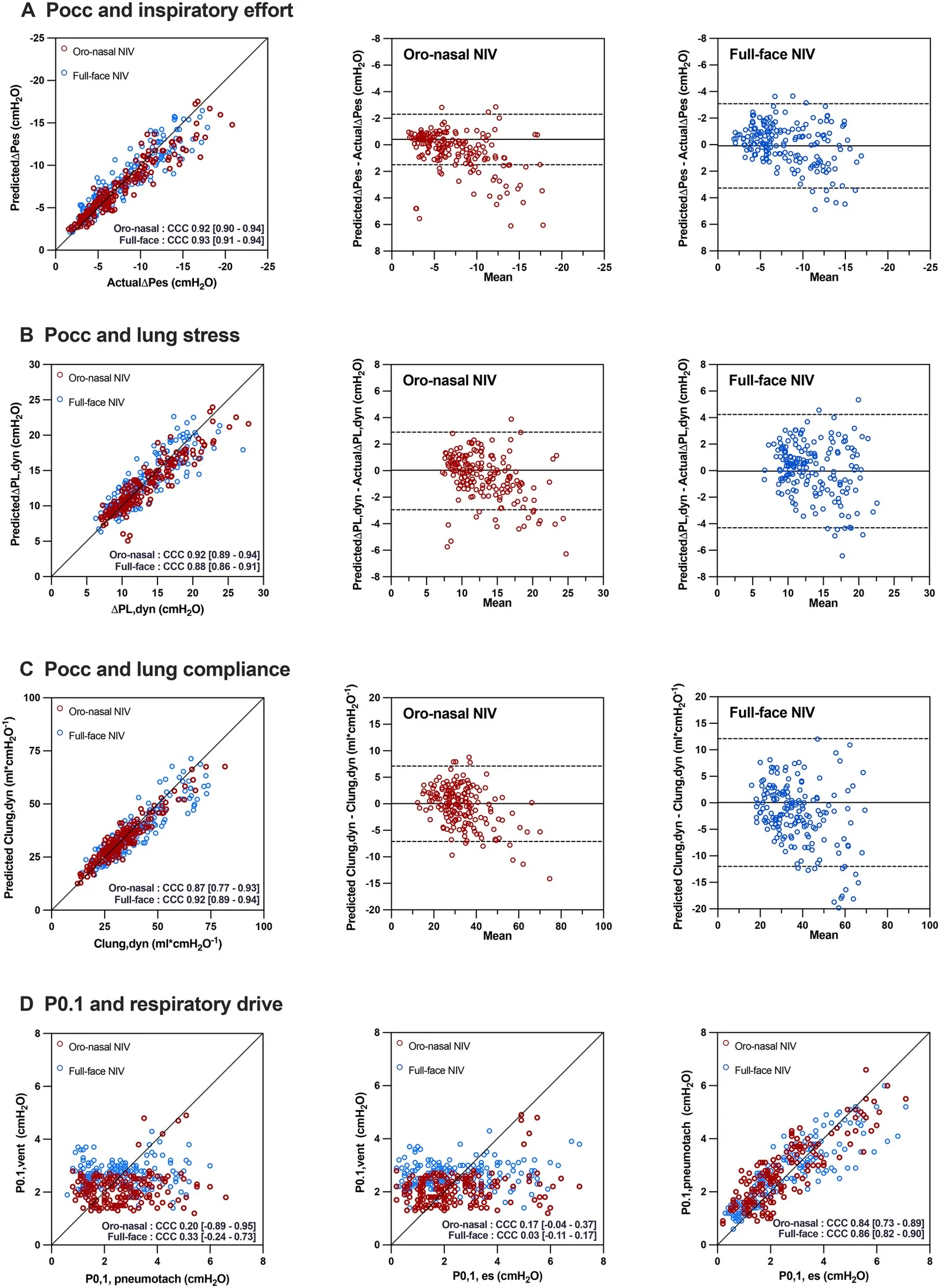
Agreement between Pocc-derived indices and reference metrics of inspiratory effort (A), lung stress (B), and lung compliance (C). Scatterplots (left) show the concordance between paired measurements; the solid line denotes the line of identity (y = x), indicating perfect agreement; each dot represents one paired measurement. Bland–Altman plots (right) were constructed using all individual out-of-sample observations generated during the repeated cross-validation procedure; the solid horizontal line indicates the mean bias and dashed lines the limits of agreement. (D) Concordance between paired measurements. Left panel: P0.1 measured by the ventilator vs P0.1 measured by the pneumotachograph. Middle panel: Ventilator-derived P0.1 vs the reference metric of respiratory drive (P0.1,es). Right panel: pneumotachograph-derived P0.1 vs P0.1,es. Abbreviations: CCC, concordance correlation coefficient; Clung,dyn, dynamic lung compliance; NIV, noninvasive ventilation; P0.1,es, esophageal pressure deflection 0.1 second after the onset of inspiratory effort; P0.1,vent, ventilator-derived airway occlusion pressure 0.1 second after the onset of inspiratory effort; Pocc, maximal negative deflection in airway pressure during end-expiratory occlusion; PL,dyn, dynamic transpulmonary driving pressure.

**Table 3:** Breathing pattern and clinical variables during oronasal and full-face NIV in patients who subsequently experienced extubation success or failure.

	**Extubation success (*n*** = **46)**		**Extubation failure (*n*** = **14)**	
	Oronasal NIV	Full-face NIV	Oronasal NIV	Full-face NIV
**Nonoccluded breaths**				
** PEEP, cm H_2_O**	5 [5-6]	5 [5-6]	5 [5-6]	5 [5-6]
** Ppeak, cm H_2_O**	11 [11-12]	11 [9-11]	11 [11-12]	11 [11-12]
** V_T_/PBW, mL/kg**	7.8 [6.9-9.5]	8.0 [7.3-9.4]	9.5 [7.9-10.2]^a^	9.0 [8.4-11.5]^a^
** Respiratory rate, breaths/min**	15 [12-20]	15 [13-20]	16 [12-25]	13 [10-19]
** Air leaks, %**	20 [17-22]	20 [16-23]	21 [14-24]	22 [19-23]
** Actual ΔPes, cm H_2_O**	−6 [−9 to −4]	−6 [−9 to −4]	−11 [−14 to −9]^b^	−13 [−13 to −9]^b^
** ΔPL, dyn, cm H_2_O**	11 [10-14]	11 [10-14]	17 [15-20]^b^	18 [15-19]^b^
** Clung, dyn**	33 [29-39]	37 [31-48]^a^	26 [20-29]^b^	26 [24-39]^b^
**Occlusion-derived parameters**				
** Pocc, cm H_2_O**	−8 [−11 to −6]	−8 [−11 to −6]	−16 [−18 to −12]^b^	−15 [−18 to −12]^b^
** Pocc, pneumotach, cm H_2_O**	−8 [−11 to −6]	−9 [−12 to −7]	−16 [−17 to −12]^b^	−16 [−17 to −14]^b^
** P0.1, es, cm H_2_O**	0.7 [0.5-1.1]	0.7 [0.4-1.1]	1.2 [1.0-1.9]^b^	1.4 [1.1-1.8]^b^
** P0.1, vent, cm H_2_O**	2.1 [1.8-2.2]	2.6 [2.5-2.8]	2.2 [2.0-2.3]^b^	2.5 [2.4-2.6]
** P0.1, pneumotach, cm H_2_O**	2.3 [1.5-3.0]	2.4 [1.5-2.9]	3.4 [2.7-4.1]^b^	3.7 [3.1-3.9]^b^
** Pplat, vent, cm H_2_O**	12 [10-14]	11 [9-13]	11 [9-13]	14 [13-14]^b^
** Pplat, 0.3 s cm H_2_O**	11 [11-12]	11 [10-12]	11 [11-12]	10 [10-11]
** Pplat, 1 s, cm H_2_O**	12 [10-14]	12 [10-13]	11 [9-13]	12 [9-13]
**Predicted values derived from occlusion**				
** Predicted ΔPes**	−6 [−8 to −4]	−7 [−9 to −5]	−11 [−12 to −9]^b^	−13 [−13 to −11]^b^
** Predicted ΔPL, dyn**	11 [10-14]	12 [11-14]	17 [14-18]^b^	18 [17-19]^b^
** Predicted Clung, dyn**	33 [29-39]	38 [31-44]	26 [21-29]^b^	26 [24-31]^b^
**Gas exchange and hemodynamics collected at the end of NIV session**				
** Arterial pH**	7.41 [7.39-7.44]	7.40 [7.37-7.43]	7.41 [7.39-7.44]	7.39 [7.35-7.43]
** PaO_2_/FiO_2_ mm Hg**	208 [174-215]	187 [154-195]	213 [185-202]	195 [172-211]
** PaCO_2_, mm Hg**	42 [37-44]	43 [40-44]	40 [38-44]^b^	39 [36-42]^b^
** Systolic arterial pressure, mm Hg**	122 [112-135]	127 [118-134]	122 [112-139]	130 [120-137]
** Diastolic arterial pressure, mm Hg**	80 [75-86]	79 [75-85]	81 [72-86]	79 [73-85]
** Heart rate, bpm**	73 [64-81]	78 [73-89]	73 [67-82]	77 [71-88]

Results are expressed as median [IQR].

Abbreviations: ΔPL,dyn, dynamic transpulmonary driving pressure; Actual ΔPes, maximal swing in esophageal pressure recorded during nonoccluded breath (reference metric for inspiratory effort); Clung,dyn, dynamic lung compliance; NIV, noninvasive ventilation; P0.1, airway occlusion pressure at 100 ms; P0.1,es, esophageal pressure drop 100 ms after the onset of inspiratory effort (reference metric for respiratory drive); Paw, airway pressure; PEEP, positive end-expiratory pressure; Pes, esophageal pressure; pneumotach, pneumotachograph-derived; Pocc, airway occlusion pressure; Ppeak, Peak airway opening pressure; Pplat, inspiratory plateau pressure recorded at 0.3 s (Pplat, 0.3 sec) and 1.0 s (Pplat, 1 sec) from the start of the end-inspiratory hold; Predicted ΔPes, K × Pocc, where Pocc is airway occlusion pressure; Predicted ΔPL,dyn, Pressure support − (K × Pocc); Predicted Clung,dyn, the ratio between expiratory tidal volume and predicted ΔPL,dyn; vent, ventilator-derived; V_T_/PBW, inspired tidal volume normalized to predicted body weight.

a Significantly different between different interfaces but in the same successful group.

b Significantly different from the success group.

### Pocc and lung mechanics

Predicted ΔPL,dyn [pressure support − (K × Pocc)] showed moderate concordance with measured ΔPL,dyn, with minimal bias (oronasal: 0.03 cm H_2_O, limits −2.9 to 2.9; full-face: −0.04 cm H_2_O, limits −4.3 to 4.2). Predicted Clung,dyn also showed moderate concordance with Clung,dyn (oronasal: bias 0.003 mL/cm H_2_O, limits −7.1 to 7.1; full-face: bias 0.05 mL/cm H_2_O, limits −12.0 to 12.1) (Figure 2B, C). As for predicted ΔPes, agreement became less precise at higher ΔPL,dyn and lower Clung,dyn values (Table S3).

### P0.1 and respiratory drive

Ventilator-derived P0.1 (P0.1,vent) had poor concordance with respiratory drive (P0.1,es) (oronasal: CCC, 0.17 [95% CI, −0.04 to 0.37]; full-face: CCC 0.03 [95% CI, −0.11 to 0.17]) (Figure 2D). Pneumotachograph-derived P0.1 showed higher concordance with P0.1,es (oronasal: CCC, 0.84 [95% CI, 0.73-0.89]; full-face: CCC, 0.86 [95% CI, 0.82-0.90]) but clinically wide limits of agreement (oronasal: bias −0.2 cm H_2_O, limits −1.7 to 1.2; full-face: bias −0.1 cm H_2_O, limits −1.7 to 1.4), indicating low precision at the individual level (Figure S3). P0.1,vent thresholds >2.7 cm H_2_O (oronasal) and >3.0 cm H_2_O (full-face) identified high drive with low sensitivity but with good specificity (>90%) (Table S4). Complementary analysis also showed no correlation between P0.1,vent and ActualΔPes/dt in both interfaces (Figure S7).

**Figure 3:**
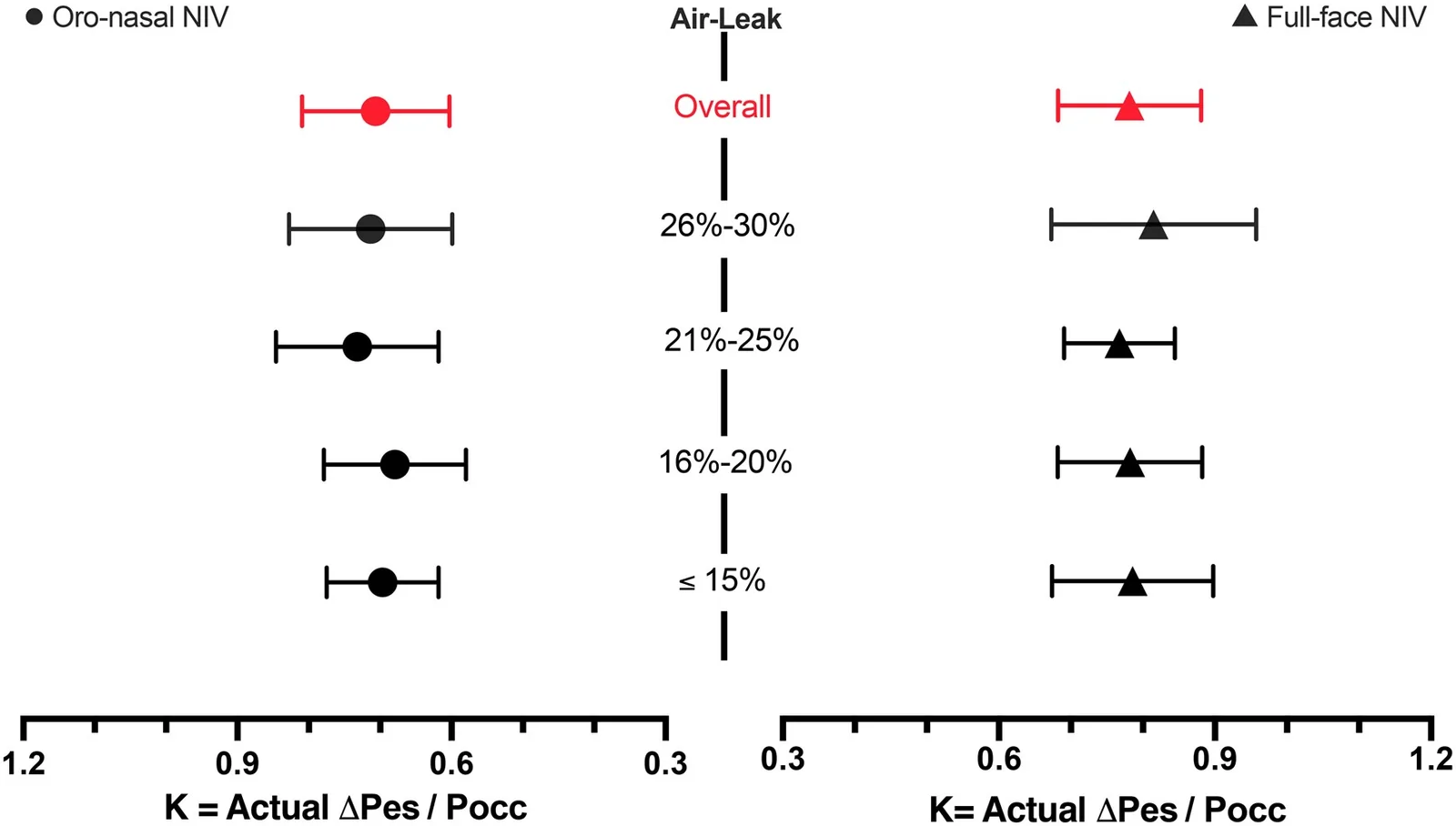
Median (IQR) conversion factor K = actual esophageal pressure swing (ΔPes) / maximal negative deflection in airway pressure during end-expiratory occlusion (Pocc) across leak categories. During oronasal noninvasive ventilation (NIV), ≤15% (*n* = 11), 16%-20% (*n* = 20), 21%-25% (*n* = 23), and 26%-30% (*n* = 6); during full-face NIV, ≤15% (*n* = 14), 16%-20% (*n* = 14), 21%-25% (*n* = 24), and 26%-30% (*n* = 8).

### Plateau pressure and lung mechanics

A stable plateau pressure tracing was obtained in 13 patients (22%) with oronasal and 6 (10%) with full-face NIV. Most occlusions showed incremental Pplat (52% and 55%), whereas decremental patterns were less frequent (26% and 35%) (Figure S5). Ventilator- and pneumotachograph-derived Pplat had high concordance (oronasal: CCC, 0.95 [95% CI, 0.94-0.96]; full-face: CCC, 0.97 [95% CI, 0.96-0.98]) but limits of agreement were wide (oronasal: bias 0.01 cm H_2_O, limits −1.7 to 1.8; full-face: bias 0.03 cm H_2_O, limits −1.6 to 1.8) (Figure S4). When Pplat was deemed reliable, static respiratory system driving pressure, neither measured with Pplat 0.3 s nor 1 s, did not show a robust correlation with ΔPL,dyn (oronasal: *r* = 0.17, *P* = .06; *r* = 0.03, *P* = .75; full-face: *r* = 0.37, *P* <.05; *r* = 0.34, *P* <.05).

**Figure 4:**
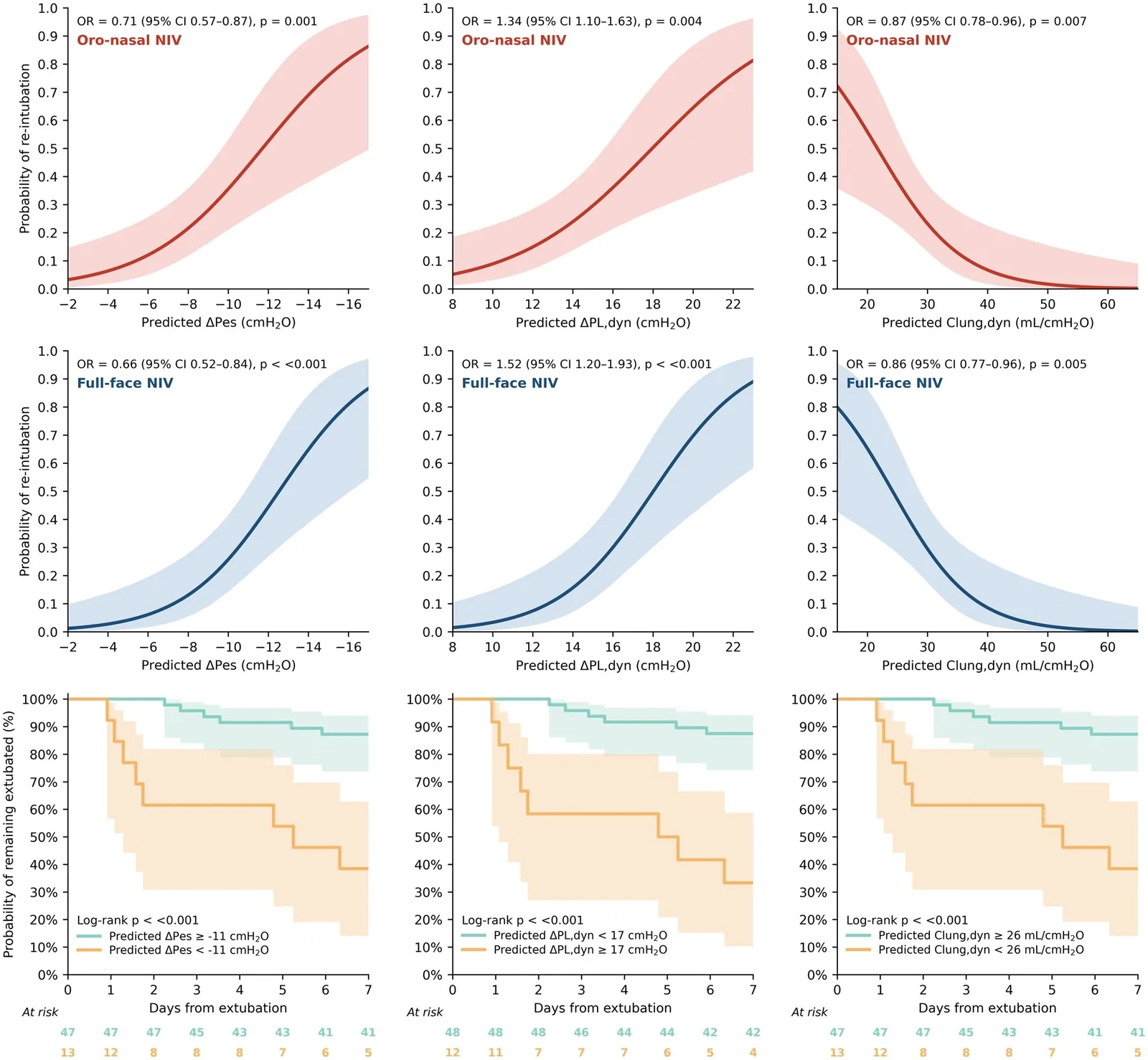
Upper panels show the probability of reintubation as a function of predicted esophageal pressure swing (ΔPes), predicted dynamic transpulmonary driving pressure (ΔPL,dyn), and predicted dynamic lung compliance (Clung,dyn) during oronasal noninvasive ventilation (NIV) (top panels) and full-face NIV (middle panels); solid curves represent the fitted logistic regression and shaded bands the 95% confidence intervals. Lower panels show the cumulative incidence of successful extubation over 7 days after extubation, stratified according to the median values observed in the failure group; solid lines denote the extubation success group and dashed lines the failure group. Shaded areas indicate 95% confidence intervals.

### NIV air leaks and occlusion-derived parameters

Median air leaks were 20% (IQR, 17%-22%) during oronasal and 21% (IQR, 17%-24%) during full-face NIV. Air leaks was not associated with Pocc (oronasal *r* = 0.22, *P* = .30; full-face *r* = 0.01, *P* = .95) and the mean conversion factor K (actual ΔPes/Pocc) remained stable across leaks levels (Figure 3), suggesting that the air leak did not meaningfully affect Pocc-derived parameters. Air leaks was also not associated with P0.1 or Pplat (Figure S6).

### Exploratory outcomes

Fourteen patients (23%) required reintubation. Patients’ characteristics according to weaning outcome are shown in Table S5; breathing pattern and clinical variables are summarized in Table 3. Among occlusion-derived parameters, more negative predicted ΔPes (oronasal: OR, 0.71 [95% CI, 0.57-0.87]; full-face: OR, 0.66 [95% CI, 0.52-0.84]), higher predicted ΔPL,dyn (oronasal: OR, 1.34 [95% CI, 1.10-1.63]; full-face: OR, 1.52 [95% CI, 1.20-1.93]), and lower predicted Clung,dyn (oronasal: OR, 0.87 [95% CI, 0.78-0.96]; full-face: OR, 0.86 [95% CI, 0.77-0.96]) were associated with subsequent reintubation (Figure 4; Table S6).

## Discussion

The results of the present multicenter physiological study conducted on patients receiving NIV through a face mask can be summarized as follows:

- End-expiratory occlusion is feasible. Pocc-derived parameters provide high concordance for inspiratory effort and moderate concordance for lung stress and lung compliance, with lower precision at extreme values; air leaks did not meaningfully affect the accuracy of these measures.
- P0.1,vent does not reliably quantify respiratory drive during NIV. This limitation seems partly related to ventilator readouts: pneumotachograph-derived P0.1 showed no systematic bias versus P0.1,es, yet the wide limits of agreement indicate poor precision. Together, these findings suggest that P0.1 has inherent limitations during NIV. Despite low overall accuracy, a high P0.1,vent (>2.7 cm H_2_O) identifies elevated drive with specificity>90%, whereas low P0.1 values are not informative.
- A stable end-inspiratory plateau pressure is measurable in only a minority of patients, limiting its use for assessing respiratory system static driving pressure.
- Higher inspiratory effort, higher dynamic transpulmonary driving pressure, and lower dynamic lung compliance, all assessed noninvasively by Pocc, are associated with subsequent reintubation.

When used in hypoxemic patients, NIV offers important advantages; however, early recognition of treatment failure is critical because it may substantially affect patient outcomes.^31-33^ At the bedside, an elevated tidal volume is one of the most recognizable warning signs of NIV failure.^34^^,^^35^ However, a high tidal volume should be better interpreted as a downstream result of the mechanistic pathway leading to lung injury progression, where excessive inspiratory effort is the primary determinant of increases in global and regional lung stress.^36^ During assisted ventilation, inspiratory effort and ventilator pressure support add to generate the transpulmonary driving pressure, which represents the elastic load applied and can be viewed as tidal volume normalized to the size of the aerated lung.^37-39^ While tidal volume often tracks this process and therefore retains prognostic value, it is less specific and can be substantially influenced by respiratory system mechanics, ventilator settings, and interface-related factors.^40^ These considerations provide a robust physiological rationale for directly monitoring inspiratory effort and transpulmonary driving pressure during NIV. Accordingly, physiological studies using esophageal pressure have shown that higher effort and transpulmonary driving pressure may predict subsequent NIV failure earlier and more accurately than absolute tidal volume.^9^^,^^11^

Esophageal manometry is the gold standard to quantify inspiratory effort and transpulmonary driving pressure during NIV, but its use is limited in clinical practice, especially in nonintubated patients.^41^ Accordingly, efforts have focused on identifying simpler, less invasive, bedside-available surrogates. In intubated patients, brief end-expiratory occlusions allow measurement of Pocc and P0.1, and end-inspiratory holds enables assessment of Pplat; these provide clinically accurate estimates of effort, respiratory drive, and lung stress without esophageal manometry.^42^^,^^43^ In our study, Pocc and its derived indices are shown to provide clinically acceptable estimates of inspiratory effort, lung stress, and compliance during NIV, with consistent performance across both oronasal and full-face interfaces. Importantly, we derived an interface-specific conversion factor to translate Pocc into predicted ΔPes, and validated this approach in a cohort, suggesting its potential generalizability. Notably, in intubated patients, the conversion factor used to estimate esophageal pressure swing from Pocc is 0.66, implying that Pocc per se overestimates Pes swing by approximately 34%. In our study this overestimation was approximately 30% with the oronasal mask (K = 0.71) and 20% with the full-face mask (K = 0.80). One possible explanation is that the face mask and upper airway add compliance to the system. As a result, part of the pressure generated during an occluded inspiratory effort may be absorbed by deformation of the interface and upper airway, rather than being fully transmitted to the airway opening. This may explain both the difference from intubated patients and the higher conversion factor observed with the full-face mask compared with the oronasal mask. Overall, these observations suggest that conversion factors derived in intubated patients should not be directly applied during NIV, as this may result in systematic underestimation of inspiratory effort.

Ventilator-derived P0.1 did not reliably quantify respiratory drive during NIV. A plausible explanation relates to patient–interface interaction: unlike in intubated patients, the first 100 ms after an occlusion are strongly influenced by mask compliance, as cushion deformability can transiently absorb patient-generated pressure, thereby damping the early airway pressure decay measured by the ventilator. Although P0.1,vent is imprecise and not accurate for quantifying drive at the individual level, it may retain some clinical usefulness as a rule-in signal: High values (eg, >3 cm H_2_O) make elevated respiratory drive likely, whereas low values cannot reliably exclude it.^44^ Limitations in point estimates for P0.1 may be attributable to ventilator readouts, since pneumotachograph-derived P0.1 showed high concordance with respiratory drive. In addition, the finding that P0.1,vent was higher than P0.1,es suggests that, during NIV, the early airway pressure signal may be influenced not only by inspiratory drive itself, but also by patient–interface interaction, mask compliance, and possibly expiratory muscle relaxation at the onset of inspiration. However, the wide limits of agreement indicate limited precision in individual point estimates, which constrains its accuracy for patient-level quantification. Notably, although the absolute dispersion of pneumotachograph-derived P0.1 was comparable to that observed for Pocc-derived estimates, its relative impact was substantially larger: Similar absolute limits of agreement translate into a much higher percentage of the expected P0.1, thereby reducing the clinical interpretability of single patient-level measurements despite an overall acceptable mean bias. Overall, our data are consistent with previous reports,^45^ suggesting that P0.1 may still be informative of respiratory drive when acquired with a high-fidelity technique (eg, pneumotachograph), whereas the additional measurement error associated with P0.1,vent may render it less reliable.

Similarly, an accurate end-inspiratory plateau pressure was obtained only in a minority of patients, limiting the clinical use of static respiratory system driving pressure and compliance during NIV. This limitation is likely multifactorial. During end-inspiratory holds, leaks and instability of the patient–interface interaction may compromise the pressure tracing. Moreover, expiratory muscle activity may further distort airway pressure during the hold, even in the presence of an apparently stable plateau.^46^^,^^47^ By contrast, the better performance of end-expiratory occlusion-derived measurements may be physiologically plausible. During end-expiratory occlusion, expiratory muscle activity may be less relevant to the interpretation of Pocc, whereas inspiratory effort generates a negative pressure deflection within the interface that may transiently improve mask sealing. Consistent with this interpretation, no association was found between the magnitude of air leaks during tidal breathing and the relationship between Pocc and inspiratory effort.

In our study, NIV-specific software was disabled during occlusion maneuvers. We recognize that this approach may limit the clinical applicability of our findings; however, this decision was driven by the need to preserve the physiological assumptions underlying occlusion-derived measurements. Since NIV algorithms are proprietary and may not be fully consistent across manufacturers, we could not rule out the possibility that leak compensation might affect the validity of occlusion-derived metrics to a variable extent depending on the ventilator used. From a practical standpoint, not all ICU ventilators allow occlusion maneuvers while NIV software is active, conversely a temporary switch from NIV mode to invasive mode appears feasible at the bedside without meaningfully altering ventilatory assistance. However, to further address this issue, we performed additional bench experiments using different ventilators and multiple combinations of PEEP and simulated inspiratory effort (Figure S8). Across all tested conditions, no clinically significant differences were observed in Pocc when occlusions were obtained with or without NIV-specific software enabled (Table S7). Although bench experiments cannot replace direct clinical validation, these additional data provide reassuring support for the robustness of our findings.

Our results have relevant clinical implications. First, bedside monitoring of inspiratory effort, transpulmonary driving pressure, and dynamic compliance may enable earlier recognition of injurious breathing patterns, thereby reducing delays in endotracheal intubation.^9^ Second, different interfaces and ventilator settings can have markedly different effects on effort and transpulmonary driving pressure.^48^ Their interaction with the individual breathing pattern, which varies across individuals, may explain why a given support strategy is effective in some patients but not in others.^49^ Pocc-derived estimates of effort and transpulmonary driving pressure may allow clinicians to individualize pressure support and PEEP in a physiology-informed manner, approximating the information that would otherwise require esophageal manometry.^50^^,^^51^ Third, inspiratory effort, transpulmonary driving pressure, and compliance may provide a pragmatic way to assess severity and stratify risk during NIV. Consistent with evidence in intubated patients,^39^^,^^52^ our exploratory outcome analyses suggest that higher effort and lung stress and lower compliance are associated with subsequent reintubation, supporting the potential prognostic value of these metrics.

Our study has limitations. First, we estimated inspiratory effort using ΔPes rather than total muscle pressure, as commonly done in intubated patients.^12^ However, estimating total muscle pressure in nonintubated patients is challenging, as it requires measurement of chest-wall elastance, which in turn relies on accurate inspiratory holds, that are inherently uncertain during NIV. Moreover, ΔPes is the effort-related metric most frequently used during NIV, and has been associated with clinically relevant outcomes in this setting.^9-11^ Second, the lack of reliability of P0.1 during NIV could theoretically be influenced by ventilator-specific algorithms. Unfortunately, we did not systematically record the ventilator model used for each patient, precluding a ventilator-specific subgroup analysis and limiting our ability to determine whether particular devices or proprietary algorithms contributed differentially to measurement error.^45^ Third, NIV was delivered using a dual-limb circuit, and NIV-specific software was disabled to avoid automatic leaks compensation; therefore, our findings may not be applicable to dedicated NIV devices that use single-limb circuits with intentional leaks setups. Fourth, the number of patients requiring reintubation was relatively small, which may have limited the statistical power of comparisons between success and failure groups. In this context, the Kaplan–Meier analysis should be interpreted as exploring the prognostic value of early physiological markers measured after extubation in relation to the subsequent risk of NIV failure. Finally, our population consisted of postextubation patients rather than patients with de novo acute hypoxemic respiratory failure. This design was necessary because accurate calibration of the esophageal balloon catheter can be reliably performed only during invasive ventilation. Although there is no clear physiological rationale to anticipate that the relationship between Pocc-derived indices and esophageal reference measurements would fundamentally differ in de novo patients, we acknowledge that using a threshold of ΔPes ≤ −10 cmH_2_O to define excessive inspiratory effort may be relatively conservative. However, several studies have shown that esophageal pressure swings above this threshold are associated with treatment failure, escalation to invasive ventilation, or potentially injurious breathing patterns.^9-11^ Importantly, our data suggest that precision decreases as inspiratory effort increases. This heteroscedasticity, however, does not preclude the use of Pocc during NIV, but rather mirrors the same pattern previously observed in intubated patients.^12^ Moreover, responses to different noninvasive supports may not differ substantially between hypoxemic and postextubation patients.^10^^,^^53^

In summary, during NIV, Pocc-derived metrics are a feasible bedside approach to quantify inspiratory effort, lung stress, and lung mechanics; P0.1 and Pplat appear to be of limited utility in this setting, although high P0.1 values may still help identify elevated drive. In our cohort, Pocc-derived indices are associated with subsequent reintubation, supporting their potential clinical relevance for early risk stratification and clinical decision-making.

## Supplementary Material

aamag293_Supplementary_Data

## Data Availability

This article has an online data supplement, which is accessible at the Supplements tab.

## References

[1] Rezoagli E , Nova A, Carteaux G, et al A clinical guide to non-invasive respiratory support in acute respiratory failure: ventilation settings, technical optimization and clinical indications. Crit Care. 2025;29:496. 10.1186/s13054-025-05730-y PMC12625427 41254751

[2] Vaporidi K , Akoumianaki E, Telias I, Goligher EC, Brochard L, Georgopoulos D. Respiratory drive in critically ill patients pathophysiology and clinical implications. Am J Respir Crit Care Med. 2020;201:20-32. 10.1164/rccm.201903-0596SO 31437406

[3] Ferreyro BL , Angriman F, Munshi L, et al Association of noninvasive oxygenation strategies with all-cause mortality in adults with acute hypoxemic respiratory failure: a systematic review and meta-analysis. JAMA. 2020;324:57-67. 10.1001/jama.2020.9524 PMC7273316 32496521

[4] Spinelli E , Mauri T, Beitler JR, Pesenti A, Brodie D. Respiratory drive in the acute respiratory distress syndrome: pathophysiology, monitoring, and therapeutic interventions. Intensive Care Med. 2020;46:606-618. 10.1007/s00134-020-05942-6 PMC7224136 32016537

[5] Grieco DL , Menga LS, Eleuteri D, Antonelli M. Patient self-inflicted lung injury: implications for acute hypoxemic respiratory failure and ARDS patients on non-invasive support. Minerva Anestesiol. 2019;85:1014-1023. 10.23736/S0375-9393.19.13418-9 30871304

[6] Henderson WR , Chen L, Amato MBP, Brochard LJ. Fifty years of research in ARDS: respiratory mechanics in acute respiratory distress syndrome. Am J Respir Crit Care Med. 2017;196:822-833. 10.1164/rccm.201612-2495CI 28306327

[7] Mauri T , Yoshida T, Bellani G, et al Esophageal and transpulmonary pressure in the clinical setting: meaning, usefulness and perspectives. Intensive Care Med. 2016;42:1360-1373. 10.1007/s00134-016-4400-x 27334266

[8] Chiumello D , Consonni D, Coppola S, Froio S, Crimella F, Colombo A. The occlusion tests and end-expiratory esophageal pressure: measurements and comparison in controlled and assisted ventilation. Ann Intensive Care. 2016;6:10. 10.1186/s13613-016-0112-1 PMC4751101 26868503

[9] Tonelli R , Fantini R, Tabbì L, et al Early inspiratory effort assessment by esophageal manometry predicts noninvasive ventilation outcome in de novo respiratory failure: a pilot study. Am J Respir Crit Care Med. 2020;202:558-567. 10.1164/rccm.201912-2512OC PMC7427381 32325004

[10] Menga LS , Delle Cese L, Rosà T, et al Respective effects of helmet pressure support, continuous positive airway pressure, and nasal high-flow in hypoxemic respiratory failure: a randomized crossover clinical trial. Am J Respir Crit Care Med. 2023;207:1310-1323. 10.1164/rccm.202204-0629OC PMC10595442 36378814

[11] Grieco DL , Menga LS, Raggi V, et al Physiological comparison of high-flow nasal cannula and helmet noninvasive ventilation in acute hypoxemic respiratory failure. Am J Respir Crit Care Med. 2020;201:303-312. 10.1164/rccm.201904-0841OC 31687831

[12] Bertoni M , Telias I, Urner M, et al A novel non-invasive method to detect excessively high respiratory effort and dynamic transpulmonary driving pressure during mechanical ventilation. Crit Care. 2019;23:346. 10.1186/s13054-019-2617-0 PMC6836358 31694692

[13] Telias I , Spadaro S. Techniques to monitor respiratory drive and inspiratory effort. Curr Opin Crit Care. 2020;26:3-10. 10.1097/MCC.0000000000000680 31764192

[14] Bellani G , Grassi A, Sosio S, Foti G. Plateau and driving pressure in the presence of spontaneous breathing. Intensive Care Med. 2019;45:97-98. 10.1007/s00134-018-5311-9 30006893

[15] Murgolo F , Grieco DL, Bertoni M, Spadaro S, Pettenuzzo T, Cardu A, et al C105-15. Non-invasive assessment of inspiratory effort in patients undergoing non-invasive ventilation: the Inspire study. Am J Respir Crit Care Med. 2026;212:aamag162.4746. 10.1093/ajrccm/aamag162.4746

[16] Akoumianaki E , Maggiore SM, Valenza F, et al The application of esophageal pressure measurement in patients with respiratory failure. Am J Respir Crit Care Med. 2014;189:520-531. 10.1164/rccm.201312-2193CI 24467647

[17] Thille AW , Gacouin A, Coudroy R, et al Spontaneous-breathing trials with pressure-support ventilation or a T-piece. N Engl J Med. 2022;387:1843-1854. 10.1056/NEJMoa2209041 36286317

[18] Thille AW , Muller G, Gacouin A, et al Effect of postextubation high-flow nasal oxygen with noninvasive ventilation vs high-flow nasal oxygen alone on reintubation among patients at high risk of extubation failure: a randomized clinical trial. JAMA. 2019;322:1465-1475. 10.1001/jama.2019.14901 PMC6802261 31577036

[19] MacIntyre NR , Cook DJ, Ely EW Jr; American College of Chest Physicians; American Association for Respiratory Care; American College of Critical Care Medicine. Evidence-based guidelines for weaning and discontinuing ventilatory support: a collective task force facilitated by the American College of Chest Physicians; the American Association for Respiratory Care; and the American College of Critical Care Medicine. Chest. 2001;120(6 Suppl):375S-395S. 10.1378/chest.120.6_suppl.375s 11742959

[20] Boles JM , Bion J, Connors A, et al Weaning from mechanical ventilation. Eur Respir J. 2007;29:1033-1056. 10.1183/09031936.00010206 17470624

[21] Fernandez MM , González-Castro A, Magret M, et al Reconnection to mechanical ventilation for 1 h after a successful spontaneous breathing trial reduces reintubation in critically ill patients: a multicenter randomized controlled trial. Intensive Care Med. 2017;43:1660-1667. 10.1007/s00134-017-4911-0 28936675

[22] Oczkowski S , Ergan B, Bos L, et al ERS clinical practice guidelines: high-flow nasal cannula in acute respiratory failure. Eur Respir J. 2022;59:2101574. 10.1183/13993003.01574-2021 34649974

[23] Bertoni M , Spadaro S, Goligher EC. Monitoring patient respiratory effort during mechanical ventilation: lung and diaphragm-protective ventilation. Crit Care. 2020;24:106. 10.1186/s13054-020-2777-y PMC7092676 32204729

[24] Soundoulounaki S , Akoumianaki E, Kondili E, et al Airway pressure morphology and respiratory muscle activity during end-inspiratory occlusions in pressure support ventilation. Crit Care. 2020;24:1-9. 10.1186/s13054-020-03169-x PMC7385937 32723356

[25] Frat JP , Grieco DL, De Jong A, et al Noninvasive respiratory supports in ICU. Intensive Care Med. 2025;51:1476-1489. 10.1007/s00134-025-08036-3 40705073

[26] Tonelli R , Protti A, Spinelli E, et al Assessing inspiratory drive and effort in critically ill patients at the bedside. Crit Care. 2025;29:339. 10.1186/s13054-025-05526-0 PMC12315345 40745324

[27] Le Stang V , Graverot M, Kimmoun A, et al Nasal high flow to modulate dyspnea in orally intubated patients. Am J Respir Crit Care Med. 2025;211:577-586. 10.1164/rccm.202405-1057OC 39680951

[28] Rittayamai N , Beloncle F, Goligher EC, et al Effect of inspiratory synchronization during pressure-controlled ventilation on lung distension and inspiratory effort. Ann Intensive Care. 2017;7:100. 10.1186/s13613-017-0324-z PMC5630544 28986852

[29] King TS , Chinchilli VM, Carrasco JL. A repeated measures concordance correlation coefficient. Stat Med. 2007;26:3095-3113. 10.1002/sim.2778 17216594

[30] Carrasco JL , Phillips BR, Puig-Martinez J, King TS, Chinchilli VM. Estimation of the concordance correlation coefficient for repeated measures using SAS and R. Comput Methods Programs Biomed. 2013;109:293-304. 10.1016/j.cmpb.2012.09.002 23031487

[31] Yoshida T , Fujino Y, Amato MBP, Kavanagh BP. Fifty years of research in ARDS. Spontaneous breathing during mechanical ventilation. risks, mechanisms, and management. Am J Respir Crit Care Med. 2017;195:985-992. 10.1164/rccm.201604-0748CP 27786562

[32] Brochard L , Slutsky A, Pesenti A. Mechanical ventilation to minimize progression of lung injury in acute respiratory failure. Am J Respir Crit Care Med. 2017;195:438-442. 10.1164/rccm.201605-1081CP 27626833

[33] Esteban A , Frutos-Vivar F, Ferguson ND, et al Noninvasive positive-pressure ventilation for respiratory failure after extubation. N Engl J Med. 2004;350:2452-2460. 10.1056/NEJMoa032736 15190137

[34] Carteaux G , Millán-Guilarte T, De Prost N, et al Failure of noninvasive ventilation for de novo acute hypoxemic respiratory failure: role of tidal volume. Crit Care Med. 2016;44:282-290. 10.1097/CCM.0000000000001379 26584191

[35] Frat JP , Ragot S, Coudroy R, et al Predictors of intubation in patients with acute hypoxemic respiratory failure treated with a noninvasive oxygenation strategy. Crit Care Med. 2018;46:208-215. 10.1097/CCM.0000000000002818 29099420

[36] Yoshida T , Nakahashi S, Nakamura MAM, et al Volume-controlled ventilation does not prevent injurious inflation during spontaneous effort. Am J Respir Crit Care Med. 2017;196:590-601. 10.1164/rccm.201610-1972OC 28212050

[37] Bellani G , Grassi A, Sosio S, et al Driving pressure is associated with outcome during assisted ventilation in acute respiratory distress syndrome. Anesthesiology. 2019;131:594-604. 10.1097/ALN.0000000000002846 31335543

[38] Grieco DL , Chen L, Dres M, Brochard L. Should we use driving pressure to set tidal volume? Curr Opin Crit Care. 2017;23:38-44. 10.1097/MCC.0000000000000377 27875410

[39] Grassi A , Bianchi I, Teggia Droghi M, et al Increased driving pressure during assisted ventilation for hypoxemic respiratory failure is associated with lower ICU survival: the ICEBERG study. Am J Respir Crit Care Med. 2025;211:2169-2179. 10.1164/rccm.202411-2146OC 40540619

[40] Amato MBPP , Meade MO, Slutsky AS, et al Driving pressure and survival in the acute respiratory distress syndrome. N Engl J Med. 2015;372:747-755. 10.1056/NEJMsa1410639 25693014

[41] De Luca D , De La Rubia S, Miselli F, et al Inspiratory effort and dynamic transpulmonary driving pressure in extremely preterm infants. Chest. 2025;169:968-976. 10.1016/J.CHEST.2025.09.119 41076064

[42] Foti G , Cereda M, Banfi G, Pelosi P, Fumagalli R, Pesenti A. End-inspiratory airway occlusion: a method to assess the pressure developed by inspiratory muscles in patients with acute lung injury undergoing pressure support. Am J Respir Crit Care Med. 1997;156:1210-1216. 10.1164/ajrccm.156.4.96-02031 9351624

[43] de Vries HJ , Tuinman PR, Jonkman AH, et al Performance of noninvasive airway occlusion maneuvers to assess lung stress and diaphragm effort in mechanically ventilated critically ill patients. Anesthesiology. 2023;138:274-288. 10.1097/ALN.0000000000004467 36520507

[44] Hilbert G , Gruson D, Portel L, Vargas F, Gbikpi-Benissan G, Cardinaud JP. Airway occlusion pressure at 0.1 s (P0.1) after extubation: an early indicator of postextubation hypercapnic respiratory insufficiency. Intensive Care Med. 1998;24:1277-1282. 10.1007/s001340050762 9885880

[45] Telias I , Junhasavasdikul D, Rittayamai N, et al Airway occlusion pressure as an estimate of respiratory drive and inspiratory effort during assisted ventilation. Am J Respir Crit Care Med. 2020;201:1086-1098. 10.1164/rccm.201907-1425OC 32097569

[46] Akoumianaki E , Vaporidi K, Stamatopoulou V, et al Gastric pressure monitoring unveils abnormal patient-ventilator interaction related to active expiration: a retrospective observational study. Anesthesiology. 2024;141:541-553. 10.1097/ALN.0000000000005071 38753985

[47] Stamatopoulou V , Akoumianaki E, Vaporidi K, Stamatopoulos E, Kondili E, Georgopoulos D. Driving pressure of respiratory system and lung stress in mechanically ventilated patients with active breathing. Crit Care. 2024;28:19. 10.1186/s13054-024-04797-3 PMC10785492 38217038

[48] Menga LS , Balzani E, Gelormini C, et al Physiological effects of noninvasive respiratory support strategies in adults with acute hypoxemic respiratory failure: a systematic review and network meta-analysis. Crit Care. 2025;29:456. 10.1186/s13054-025-05670-7 PMC12560409 41152956

[49] Luca Grieco D , Menga L, Cesarano M, et al Phenotypes of patients with COVID-19 who have a positive clinical response to helmet noninvasive ventilation. Am J Respir Crit Care Med. 2022;205:360-365. 10.1164/rccm.202105-1212LE PMC8887003 34788583

[50] Grieco DL , Munshi L, Piquilloud L. Personalized noninvasive respiratory support for acute hypoxemic respiratory failure. Intensive Care Med. 2023;49:840-843. 10.1007/s00134-023-07048-1 PMC10140708 37115260

[51] Cutuli SL , Grieco DL, Michi T, et al Personalized respiratory support in ARDS: a physiology-to-bedside review. J Clin Med. 2023;12:4176. 10.3390/jcm12134176 PMC10342961 37445211

[52] Dianti J , Lovblom LE, Iftikhar MA, et al Association of respiratory drive and effort with mortality and time to discharge in patients on mechanical ventilation in Canada: a longitudinal, prospective, registry-based cohort study. Lancet Respir Med. 2025;13:1087-1095. 10.1016/S2213-2600(25)00297-8 40983065

[53] Arrive F , Le Pape S, Bruhn A, et al Physiological comparison of noninvasive ventilation and high-flow nasal oxygen on inspiratory efforts and tidal volumes after extubation: a randomized crossover trial. Crit Care. 2025;29:185. 10.1186/s13054-025-05366-y PMC12060563 40341100

