# Supplementary material for: Airway occlusions to measure inspiratory effort, respiratory drive, and lung mechanics during noninvasive ventilation
Source: Am J Respir Crit Care Med. 2026 Jun 13;212(11):2756–68. doi: 10.1093/ajrccm/aamag293 (PMC13721124; doi:10.1093/ajrccm/aamag293)
Supplement: Supplementary file 1 [file aamag293_supplementary_data.zip › OS_R2.docx]

***ONLINE DATA***

***SUPPLEMENT***

**Airway Occlusions to Measure Inspiratory Effort, Respiratory Drive, and Lung Mechanics During Noninvasive Ventilation**

Francesco Murgolo*^1,2,3^; Domenico Luca Grieco*^4,5^; Rossana Soloperto^1,2^; Michele Bertoni^6,7^; Savino Spadaro^8^; Nicola Bartolomeo^9^; Tommaso Pettenuzzo^10^; François Beloncle^11,12^; Delphine Venkatapen^11,13^; Alessandro Cardu^4,5^; Tommaso Rosà^4,5^; Chiara Iacovelli^4,5^, Paolo Navalesi^10,14^; Salvatore M. Maggiore^15^; Ewan C. Goligher^3^; V. Marco Ranieri^1,2^; Lorenzo del Sorbo^3^; Massimo Antonelli*^4^**^,^**^5^; Salvatore Grasso* ^1,2^.

*These authors equally contributed to the manuscript

**TABLE OF CONTENTS**

**Methods**……...……………………..……………………………………………………3

**Table E1**………………………...…..………………………………………………..…12

**Table E2**………………………...…..…………………………………………………..13

**Table E3**………………………...…..………………………………………………..…14

**Table E4**………………………...…..…………………………………………………..15

**Table E5**………………………...…..…………………………………………………..17

**Figure E1**……………………...…..………………..…………………………………..18

**Figure E2**……………………...…..………………..…………………………………..19

**Figure E3**……………………...…..…………………..………………………………..20

**Figure E4**……………………...…..……………………..…………………………..…21

**Figure E5**……………………...…..……………………..…………………………..…22

**Figure E6**……………………...…..……………………..…………………………..…23

**Bench assessment (including Table E7 and Figure E8)**..……………….…………...26

**METHODS**

**Study Procedure**

Study procedures are summarized in **Figure E1**. After completion of the spontaneous breathing trial, patients who met eligibility criteria were reconnected to the ventilator and re-ventilated for 1 hour using pressure support and their clinical ventilator settings to prevent fatigue (1). At the beginning of the study protocol, an esophageal balloon catheter (Nutrivent, Sidam, Mirandola, Italy) was inserted if not already in place; the esophageal balloon was inflated with 4 ml of air, which is a non-stress filling volume that ensures stable pressure transmission across a wide operating range for the Nutrivent catheter (2, 3). In all patients, the Pes signal was verified according to guideline recommendations, and calibration was confirmed using a dynamic occlusion test (4). Catheter calibration was considered adequate, and patients were included when the ratio of changes in airway pressure to changes in esophageal pressure during an occluded breath was between 0.8 and 1.2. Subsequently, patients were extubated and NIV was immediately initiated. NIV was delivered using a gas-compressed mechanical ventilator connected via a dual-limb circuit and a heat-and-moisture exchanger. NIV was delivered in pressure support mode; for study purposes, NIV-specific ventilator software was not activated (invasive ventilation mode), thereby disabling air leak compensation. NIV was initially set with positive end-expiratory pressure (PEEP) of 5–7 cmH_2_O and pressure support of 5 cmH_2_O, then titrated to achieve a tidal volume of 6–8 mL/kg predicted body weight. FiO_2_ was adjusted to maintain SpO_2_ between 92 and 96%. Pressurization time was set at 50–100 ms and expiratory trigger sensitivity at 20–50% of peak inspiratory flow.

All patients underwent two 1-hour NIV sessions in randomized order (computer-generated random sequence), using two different interfaces (oro-nasal and full-face masks), both equipped with a dedicated nasogastric-tube port (Dimar Zero, HMC GROUP, Mirandola, Italy). These interfaces were specifically selected to allow passage of the nasogastric tube while minimizing additional leaks and helping secure the tube in position during NIV. During each NIV session, at least three non-consecutive end-expiratory and end-inspiratory occlusion maneuvers were performed. Between the two NIV sessions, patients received high-flow nasal oxygen for 1 hour; flow and temperature were adjusted to optimize comfort and FiO_2_ was titrated to maintain SpO_2_>92%. Throughout all study procedures, sedation was avoided or limited to minimal-dose dexmedetomidine, targeting a Richmond Agitation and Sedation Scale (RASS) of 0 to +2. Dosage was not modified throughout study procedures. After completion of the protocol, patients were managed with alternating high-flow nasal oxygen and NIV, for at least 48 hours, according to clinical practice (5). The decision to re-intubate the patients in case of post-extubation distress was made by attending physicians, who were blinded to study measurements.

**Measurements**

Patients’ demographics and most relevant clinical characteristics were collected at study entry. Airway flow, measured with Fleisch-type pneumotachograph (No. 2, Metabo, Lausanne, Switzerland) placed between the NIV mask and the dual-limb circuit, Pes and airway pressure (Paw) were continuously recorded at 200 Hz and stored for offline analysis through a dedicated software (ICU Lab, Kleistek, Bari, Italy).

For each patient, 155–222 non-occluded breaths, three end-expiratory occlusions, and three end-inspiratory occlusions were analyzed. Peak airway pressure and PEEP were measured from the Paw signal. Tidal volume (V_T_) was measured as the digital integral of the expiratory flow. Pocc was defined as the maximal negative deflection in airway pressure during the first breath with occluded circuit (6), and P0.1 was defined as the airway pressure drop in the initial 100 ms of the occluded breath (7, 8). Pplat was measured both at 0.3 and 1.0 second from the onset of the end-inspiratory occlusion (Pplat,0.3s and Pplat**,**1s respectively). Pplat stability was defined as a difference of ≤±1 cmH_2_O between the two values; otherwise, Pplat was classified as unstable (incremental or decremental Pplat) (9) (**Figure 1**). Airway driving pressure (Dp) was quantified by subtracting PEEP from Pplat (9). In addition to offline measurements derived from pneumotachograph, Pocc, P0.1, and Pplat were also recorded directly from the ventilator screen. Due to technical limitations, ventilator-measured Pplat value was collected only at the end of the inspiratory hold.

Inspiratory effort was calculated as the negative esophageal pressure swing during non-occluded breaths (actual ΔPes) as not affected by air-leak (10). Actual ΔPes was averaged breath-by-breath over a 5-min period of stable breathing immediately before the occlusion maneuvers. Patient’s respiratory drive was referenced to the Pes drop 100 ms after the onset of inspiratory effort during end-expiratory occlusion (P0.1,es) (8, 11). As complementary analysis, respiratory drive was also assessed by the maximal rate of esophageal pressure decrease during inspiration (ΔPes/dt) measured on non-occluded breaths. Transpulmonary pressure was obtained by subtracting Pes from Paw (4). Lung stress was referenced to dynamic transpulmonary driving pressure (ΔPL,dyn), defined as the positive swing in transpulmonary pressure from the onset of inspiration to its peak value (6, 12). Dynamic lung compliance (Clung,dyn) was computed as the ratio between expiratory tidal volume and ΔPL,dyn (11). For all calculations, the beginning of inspiration was identified at the point of initial Pes decay (13). Air leaks were measured as the relative difference between inspired and expired tidal volume and were averaged over a 5-minute observation period to reduce variability. At the end of each NIV session, arterial blood gases, heart rate and arterial pressure were recorded.

**Statistical Analysis**

Descriptive statistics are presented as median [interquartile range] or number (percentage), as appropriate. Normality of continuous variables was assessed using the Shapiro–Wilk test. Intra-patient variability was quantified using the coefficient of variation (CV%) and the intraclass correlation coefficient (ICC) (**Table E1**). Between-group comparisons were performed using the chi-square test or the Mann–Whitney U test, as appropriate. Within-group comparisons were conducted using the Wilcoxon signed-rank test. All tests were two-tailed, and a p-value <0.05 was considered statistically significant.

*Derivation of inspiratory effort and lung stress from occlusion pressure*

To calculate inspiratory effort and lung stress from occlusion pressure (Pocc), an internal cross-validation procedure was performed and repeated 100 times. At each iteration, patients were randomly assigned to a derivation cohort (one third of the population) and a validation cohort (two thirds). In the derivation cohort, a proportionality constant was estimated as K = (actual ΔPes /Pocc) using linear mixed-effects models with patient as a random effect to account for repeated measurements and applied it in the validation set to compute predicted ΔPes = (K × Pocc) and predicted ΔPL,dyn =[Pressure support - (K × Pocc)] (6). The assumption of linearity between Pocc and measured ΔPes was evaluated prior to model finalization through graphical inspection of conditional residuals plotted against Pocc, with locally weighted smoothing (LOESS) curves used to identify potential departures from linearity. In addition, residual diagnostics included visual inspection of Q–Q plots as well as distribution and probability plots of the conditional residuals. These diagnostic evaluations did not reveal systematic curvature or other patterns indicative of non-linearity. The results were consistent across both interface types (oronasal and full-face), supporting the assumption of a linear relationship between Pocc and measured ΔPes in the mixed-effects models (**Figure S1**) For all analyses, actual ΔPes, predicted ΔPes and Pocc were expressed with their physical signs (i.e., as negative deflections relative to atmospheric/airway baseline). To compute all occlusion-derived parameters we used Pocc measured by ventilator.

*Feasibility and accuracy of occlusion-derived measurements*

Feasibility was the association and agreement between ventilator-derived Pocc, P0.1 and Pplat and their corresponding values measured with pneumotach; for Pplat, feasibility also required a stable pressure tracing during the inspiratory hold (as a difference of ≤±1 cmH_2_O between Pplat**,**1s and Pplat,0.3s). Accuracy was evaluated by comparing occlusion-derived parameters kept from the ventilator with reference measures of inspiratory effort, respiratory drive, lung stress and mechanics derived from Pes tracing.

*Association and agreement analyses*

Associations between predicted and measured values were assessed using mixed-effects models to account for repeated measurements within patients: patients were included as random effects and time included as a fixed effect. Agreement between methods was evaluated using the concordance correlation coefficient (CCC) for longitudinal repeated measurements. The CCC was estimated using the U-statistics methodology described by King et al. (14), which accounts for within-subject correlation across repeated time points and provides an unbiased assessment of agreement between two methods in balanced repeated-measures data. This approach is particularly appropriate for small to moderate sample sizes and for data that may deviate from normality, as described by Carrasco et al. (15). Confidence intervals and standard errors were derived from the asymptotic variance of the estimator.

Graphical agreement was assessed using Bland–Altman analysis adapted for repeated measurements. Limits of agreement were estimated using linear mixed-effects models with patient-specific random intercepts, allowing separation of within-patient variability from between-patient bias. As no proportional error was detected, limits of agreement were assumed to be constant across the measurement range and were therefore represented as horizontal lines. The mean bias was estimated as the fixed intercept of the mixed-effects model, while the within-patient standard deviation was derived from the residual variance. The 95% limits of agreement were calculated as the mean bias ±1.96 times the within-patient standard deviation. Bland–Altman plots were constructed using all individual out-of-sample observations generated during the repeated cross-validation procedure.

*Discrimination for excessive inspiratory effort and high respiratory drive*

Excessive inspiratory effort was defined a priori as actualΔPes ≤ −10 cmH₂O (16, 17). High inspiratory drive was defined a priori as P0.1,es > 3 cmH₂O (8). Discrimination of predicted ΔPes for excessive effort was assessed in the validation cohorts using receiver operating characteristic (ROC) curve analysis. Sensitivity, specificity, and the Youden index were calculated at each iteration; summary estimates and 95% confidence intervals were obtained by nonparametric bootstrapping. Discrimination of P0.1 for high respiratory drive was assessed in the entire cohort (60 patients) using ROC analysis as well.

*Complementary analysis for respiratory drive*

As a complementary analysis, the association between ΔPes/dt and P0.1,es was assessed, and the same approach was applied to ventilator-derived and pneumotachograph-derived P0.1 (Figure E7). Mixed-effects models were used with patient as random effect and time as fixed effect, separately by interface.

*Clinical outcomes*

Extubation failure was defined as re-intubation due to acute respiratory failure within 7 days after extubation. For each physiological variable, patient-level values were summarized as the mean across the three study measurements. The association between each variable and extubation failure was assessed using univariable logistic regression, and analyses were performed separately according to the NIV interface. Time to re-intubation was summarized using Kaplan–Meier curves, with patients dichotomized according to the median value of Predicted ΔPes, Predicted ΔPL,dyn and Predicted Clung,dyn observed in extubation failure group; survival curves were compared using the log-rank test.

*Sample size*

Sample size was determined a priori for the primary objective, namely to define the number of patients required in the derivation cohort to estimate the proportionality coefficient used to convert Pocc into actual Pes ( K= actual ∆Pes/Pocc) as described elsewhere (6). Assuming a correlation coefficient of 0.70 between Pocc and ΔPes (α=0.05, power=80%) (6). The required sample size was 16 patients; to improve the precision and robustness of the estimate, however, given the uncertainty regarding the potential impact of air-leak on the stability of K we increased the derivation cohort to 20 patients. The validation cohort size was then set a priori at twice the derivation cohort (n=40) to maximize the number of out-of-sample observations for performance assessment, resulting in a total sample of 60 patients.

*Supports*

All analyses were performed using SAS version 9.4 (SAS Institute, Cary, NC).

*Figure S1.*

*References*

1.	Fernandez MM, González-Castro A, Magret M, Bouza MT, Ibañez M, García C, Balerdi B, Mas A, Arauzo V, Añón JM, Ruiz F, Ferreres J, Tomás R, Alabert M, Tizón AI, Altaba S, Llamas N, Fernandez R. Reconnection to mechanical ventilation for 1 h after a successful spontaneous breathing trial reduces reintubation in critically ill patients: a multicenter randomized controlled trial. *Intensive Care Med* 2017;43:1660–1667.

2.	Mojoli F, Iotti GA, Torriglia F, Pozzi M, Volta CA, Bianzina S, Braschi A, Brochard L. In vivo calibration of esophageal pressure in the mechanically ventilated patient makes measurements reliable. *Crit Care* 2016;20:.

3.	Mojoli F, Chiumello D, Pozzi M, Algieri I, Bianzina S, Luoni S, Volta CA, Braschi A, Brochard L. Esophageal pressure measurements under different conditions of intrathoracic pressure. An in vitro study of second generation balloon catheters. *Minerva Anestesiol* 2015;81:855–864.

4.	Akoumianaki E, Maggiore SM, Valenza F, Bellani G, Jubran A, Loring SH, Pelosi P, Talmor D, Grasso S, Chiumello D, Gué Rin C, Patroniti N, Ranieri VM, Gattinoni L, Nava S, Terragni PP, Pesenti A, Tobin M, Mancebo J, Brochard L. The application of esophageal pressure measurement in patients with respiratory failure. *Am J Respir Crit Care Med* 2014;189:520–531.

5.	Oczkowski S, Ergan B, Bos L, Chatwin M, Ferrer M, Gregoretti C, Heunks L, Frat JP, Longhini F, Nava S, Navalesi P, Ugurlu AO, Pisani L, Renda T, Thille AW, Winck JC, Windisch W, Tonia T, Boyd J, Sotgiu G, Scala R. ERS clinical practice guidelines: high-flow nasal cannula in acute respiratory failure. *Eur Respir J* 2022;59:.

6.	Bertoni M, Telias I, Urner M, Long M, Del Sorbo L, Fan E, Sinderby C, Beck J, Liu L, Qiu H, Wong J, Slutsky AS, Ferguson ND, Brochard LJ, Goligher EC. A novel non-invasive method to detect excessively high respiratory effort and dynamic transpulmonary driving pressure during mechanical ventilation. *Crit Care* 2019;23:.

7.	Alberti A, Gallo F, Fongaro A, Valenti S, Rossi A. P0.1 is a useful parameter in setting the level of pressure support ventilation. *Intensive Care Med* 1995;21:547–553.

8.	Le Stang V, Graverot M, Kimmoun A, Niérat MC, Decavèle M, Similowski T, Demoule A, Dres M. Nasal High Flow to Modulate Dyspnea in Orally Intubated Patients. *Am J Respir Crit Care Med* 2025;211:577–586.

9.	Soundoulounaki S, Akoumianaki E, Kondili E, Pediaditis E, Prinianakis G, Vaporidi K, Georgopoulos D. Airway pressure morphology and respiratory muscle activity during end-inspiratory occlusions in pressure support ventilation. *Crit Care* 2020;24:1–9.

10.	Frat JP, Grieco DL, De Jong A, Gibbs K, Carteaux G, Roca O, Lemiale V, Piquilloud L, Rittayamai N, Pisani L, Hernandez G, Thille AW. Noninvasive respiratory supports in ICU. *Intensive Care Med* 2025;51:1476–1489.

11.	Tonelli R, Protti A, Spinelli E, Grieco DL, Yoshida T, Jonkman AH, Akoumianaki E, Telias I, Docci M, Rodrigues A, Perez J, Piquilloud L, Beitler J, Liu L, Roca O, Pisani L, Goligher E, Carteaux G, Bellani G, Clini E, Zhou JX, Grasselli G, Jaber S, Demoule A, Talmor D, Heunks L, Brochard L, Mauri T. Assessing inspiratory drive and effort in critically ill patients at the bedside. *Crit Care* 2025;29:.

12.	Bellani G, Grassi A, Sosio S, Foti G. Plateau and driving pressure in the presence of spontaneous breathing. *Intensive Care Med* 2019;45:97–98.

13.	Rittayamai N, Beloncle F, Goligher EC, Chen L, Mancebo J, Richard JCM, Brochard L. Effect of inspiratory synchronization during pressure-controlled ventilation on lung distension and inspiratory effort. *Ann Intensive Care* 2017;7:.

14.	King TS, Chinchilli VM, Carrasco JL. A repeated measures concordance correlation coefficient. *Stat Med* 2007;26:3095–3113.

15.	Carrasco JL, Phillips BR, Puig-Martinez J, King TS, Chinchilli VM. Estimation of the concordance correlation coefficient for repeated measures using SAS and R. *Comput Methods Programs Biomed* 2013;109:293–304.

16.	Tonelli R, Fantini R, Tabbì L, Castaniere I, Pisani L, Pellegrino MR, Casa G Della, D’Amico R, Girardis M, Nava S, Clini EM, Marchioni A. Early inspiratory effort assessment by esophageal manometry predicts noninvasive ventilation outcome in de novo respiratory failure: A pilot study. *Am J Respir Crit Care Med* 2020;202:558–567.

17.	Menga LS, Delle Cese L, Rosà T, Cesarano M, Scarascia R, Michi T, Biasucci DG, Ruggiero E, Dell’Anna AM, Cutuli SL, Tanzarella ES, Pintaudi G, De Pascale G, Sandroni C, Maggiore SM, Grieco DL, Antonelli M. Respective Effects of Helmet Pressure Support, Continuous Positive Airway Pressure, and Nasal High-Flow in Hypoxemic Respiratory Failure: A Randomized Crossover Clinical Trial. *Am J Respir Crit Care Med* 2023;207:1310–1323.

**Table E1. Intra-patient variability analysis for repeated measurement.**

|  | **Oro-nasal NIV** | | **Full-face NIV** | |
| --- | --- | --- | --- | --- |
|  | **CV (%)**  **Median [IQR]** | **ICC**  **Median [IQR]** | **CV (%)**  **Median [IQR]** | **ICC**  **Median [IQR]** |
| *Non-occluded breaths* |  |  |  |  |
| PEEP, cmH_2_O | 4.1 [2.7 – 5.7] |  | 4.1[2.7 – 5.8] |  |
| Ppeak, cmH_2_O | 2.1 [1.2 – 3.6] |  | 1.7[1.3 – 3.4] |  |
| V_T_ / PBW, mL/kg | 7.1 [4.5 – 8.6] |  | 8.1[5.5 – 13.7] |  |
| Respiratory Rate, breaths/min | 7.4 [4.07 – 10.1] |  | 7.9[5.0 – 10.8] |  |
| Actual ΔPes, cmH_2_O | -5.5 [-9.9 – -3.2] |  | -7.2[-14.3 – -4.7] |  |
| ΔPL Dyn, cmH_2_O | 13.2 [7.2 – 17.4] |  | 13.6[7.5 – 18.3] |  |
| *Occluded-derived parameters* |  |  |  |  |
| Pocc, |  | 0.94 [0.92-0.96] |  | 0.82 [0.76-0.88] |
| Pocc, pneumotach |  | 0.95 [0.92-0.97] |  | 0.91 [0.79-0.94] |
| P0.1, es |  | 0.86 [0.74-0.91] |  | 0.84 [0.71-0.88] |
| P0.1, vent, |  | 0.81 [0.73-0.92] |  | 0.77 [0.71-0.82] |
| P0.1, pneumotach, |  | 0.93 [0.90-0.95] |  | 0.88 [0.79-0.92] |
| Pplat, vent, |  | 0.77 [0.75-0.86] |  | 0.74 [0.62-0.81] |
| Pplat,0.3s |  | 0.76 [0.53-0.82] |  | 0.64 [0.36-0.60] |
| Pplat,1s, |  | 0.81 [0.79-0.89] |  | 0.72 [0.56-0.76] |
|  |  |  |  |  |

Abbreviation: NIV: noninvasive ventilation; Paw: airway pressure; PEEP: positive end-expiratory pressure; Ppeak: Peak airway opening pressure; V_T_/PBW: inspired tidal volume normalized to predicted body weight; Pes: esophageal pressure; Actual ΔPes: maximal swing in esophageal pressure recorded during non-occluded breath (reference metric for inspiratory effort); P0.1,es: Pes drop 100 ms after the onset of inspiratory effort (reference metric for respiratory drive);PL: transpulmonary pressure; ΔPL,dyn: Dynamic transpulmonary driving pressure; Pocc: airway occlusion pressure; P0.1: airway occlusion pressure at 100 ms; Pplat: Inspiratory plateau pressure recorded at 0.3 s (Pplat,0.3s) and 1.0 s (Pplat,1s) from the start of the end-inspiratory hold.

**Table E2: Discriminative performance of predicted ∆Pes for detecting excessive inspiratory effort**

| **Interface** | **Parameter** | **Operating Definition of Abnormal Condition** | **AUC (95% CI)** | **Cut-off of Predicted Value** | **Sensitivity (95% CI)** | **Specificity (95% CI)** |
| --- | --- | --- | --- | --- | --- | --- |
| Oro-nasal NIV | Predicted ∆Pes | Actual∆Pes ≤ −10 cmH_2_O | 0.98 (0.97–0.99) | Predicted ∆Pes< −8 cm H_2_O | 1.00 (0.93–1.00) | 0.84 (0.77–0.90) |
|  |  |  |  | Predicted ∆Pes < −9 cm H_2_O | 0.90 (0.79–96) | 0.95 (0.90–0.98) |
|  |  |  |  | Predicted ∆Pes < −10 cm H_2_O | 0.78 (0.65–87) | 0.99 (0.96–1.00) |
| Full-face NIV | Predicted ∆Pes | Actual∆Pes ≤ −10 cmH_2_O | 0.97 (0.96–0.99) | Predicted ∆Pes< −8 cm H_2_O | 1.00 (0.93–1.00) | 0.67 (0.59–0.74) |
|  |  |  |  | Predicted ∆Pes < −9 cm H_2_O | 0.98 (0.89–1.00) | 0.88 (0.82–0.93) |
|  |  |  |  | Predicted ∆Pes < −10 cm H_2_O | 0.84 (0.72–91) | 0.95 (0.90–0.98) |

Abbreviation: NIV: noninvasive ventilation; ActualΔPes: maximal swing in esophageal pressure recorded during non-occluded breath (reference metric for inspiratory effort); PredictedΔPes = K x Pocc, where Pocc is airway occlusion pressure

**Table E3. Proportional limits of agreement across the range of inspiratory effort**

|  | **Parameter** | **Q1** | **Q2** | **Q3** |
| --- | --- | --- | --- | --- |
| Oro-nasal NIV | Predicted∆Pes | −4.4 [−1.8;0.9] | −6.2 [−2.2;1.4] | −9.8 [−3.1;2.3] |
|  | Predicted∆PL,dyn | 10 [-2.3;2.4] | 12.2 [-2.8;2.9] | 15.7 [-3.6;3.6] |
|  | Predicted Clung,dyn | 26.4 [-6.0;6.0] | 31.4 [-7.1;7.1] | 37.6 [-8.5;8.5] |
|  |  |  |  |  |
| Full-face NIV | Predicted∆Pes | −5.3 [−2.3;2.5] | −6.9 [−3.1;3.3] | −11.3 [−4.9;5.1] |
|  | Predicted∆PL,dyn | 10.3 [-3.5;3.4] | 12.5 [-4.2;4.1] | 16.6 [-5.6;5.5] |
|  | Predicted Clung,dyn | 27.8 [-9.3;9.4] | 35.7 [-11.9;12] | 44.7 [-14.9;15.0] |

Data shows as mean [LoA]

**Table E4: Discriminative performance of P0.1 for detecting excessive respiratory drive**

| **Interface** | **Parameter** | **Operating Definition of Abnormal Condition** | **AUC (95% CI)** | **Best Cut-off (Youden)** | **Sensitivity (95% CI)** | **Specificity (95% CI)** |
| --- | --- | --- | --- | --- | --- | --- |
| Oro-nasal NIV | P0.1,vent | P0.1,es > 3 cmH_2_O | 0.54 (0.44–0.64) | > 2.7 cmH_2_O | 0.18  (0.07-0.28) | 0.96  (0.92-0.99) |
|  | P0.1,pneumotach | P0.1,es > 3 cmH_2_O | 0.92 (0.88–0.95) | > 2.8 cmH_2_O | 0.92  (0.84-0.98) | 0.82  (0.76-0.88) |
| Full-face NIV | P0.1,vent | P0.1,es > 3 cmH_2_O | 0.52 (0.42–0.60) | > 3 cmH_2_O | 0.24  (0.14-0.35) | 0.85  (0.79-0.92) |
|  | P0.1,pneumotach | P0.1,es > 3 cmH_2_O | 0.94 (0.90–0.97) | > 2.8 cmH_2_O | 0.92  (0.85-0.98) | 0.85  (0.79-0.93) |

**Table E5. Demographics and baseline characteristics of Extubation success and failure group**

|  | **Factor** | **Extubation Success**  **(n=46)** | **Extubation Failure**  **(n=14)** | **P value** |
| --- | --- | --- | --- | --- |
|  | Age, years | 64 [57, 72] | 64 [58, 71] | 0.739 |
|  | Men, nº (%) | 34 (73.9) | 9 (64.3) | 0.511 |
|  | APACHE II ^a^, | | | |
|  | At ICU admission | 16 [13, 20] | 18 [17, 20] | 0.103 |
|  | On day of extubation | 11 [9, 16] | 13 [10, 16] | 0.574 |
|  | Length of MV before extubation, days | 5 [4, 6] | 4 [4, 6] | 0.469 |
|  | Body mass index ^b^ | 27 [25, 29] | 28 [26, 29] | 0.882 |
|  | Predicted Body Weight, Kg^c^ | 62 [54, 70] | 61 [54, 68] | 0.841 |
|  | RASS | 1 [0, 2] | 1 [0, 2] | 0.993 |
| Diagnosis at admission, n (%) | | | |  |
| Primary respiratory failure | | 25 (54.3) | 12 (85.7) | 0.058 |
| Medical | | 12 (26.1) | 0 (0.0) | 0.052 |
| Postoperative Care | | 9 (19.6) | 2 (14.3) | 1.00 |
|  | Comorbidities, n (%) | | | |
|  | Arterial hypertension | 12 (26.1) | 6 (42.9) | 0.319 |
|  | Neurologic disease | 9 (19.6) | 3 (21.4) | 1.000 |
|  | Diabetes mellitus | 13 (28.3) | 4 (28.6) | 1.000 |
|  | Renal disease | 8 (17.4) | 3 (21.4) | 0.707 |
|  | Liver disease | 8 (17.4) | 5 (35.7) | 0.159 |
|  | High-risk factors for reintubation, n (%) | | | |
|  | Age >65 years | 20 (43.5) | 7 (50.0) | 0.763 |
|  | Heart failure as the primary indication for mechanical ventilation | 12 (26.1) | 1 (7.1) | 0.264 |
|  | Moderate-to-severe COPD | 0 (0.0) | 0 (0.0) |  |
|  | APACHE II on  extubation day > 12 | 19 (41.3) | 9 (64.3) | 0.220 |
|  | Body mass index >30 | 4 (9) | 1 (7) | 0.873 |
|  | inadequate cough reflex | 7 (15.2) | 2 (14.3) | 1.000 |
|  | Prolonged mechanical  ventilation ^d^ | 4 (8.7) | 0 (0.0) | 0.564 |
|  | n>2 of comorbidities ^e^ | 33 (71.7) | 8 (57.1) | 0.338 |
|  | Baseline ventilation, oxygenation and hemodynamics before SBT | | | |
|  | PEEP, cmH_2_O | 7 [6, 8] | 7 [6, 8] | 0.926 |
|  | PS, cmH_2_O | 7 [6, 8] | 7 [6, 8] | 0.611 |
|  | V_T_/PBW | 7.3 [6.9, 7.5] | 7.1 [6.8, 7.4] | 0.399 |
|  | FiO_2_ | 0.35 [0.30, 0.40] | 0.35 [0.30, 0.40] | 0.730 |
|  | Respiratory rate, bpm | 21 [19, 25] | 18 [17, 22] | 0.118 |
|  | Arterial pH | 7.41 [7.38, 7.43] | 7.39 [7.39, 7.42] | 0.673 |
|  | PaO_2_, mm Hg | 97 [87, 105] | 99 [93, 107] | 0.618 |
|  | PaCO_2_, mm Hg | 41 [36, 46] | 43 [38, 47] | 0.563 |
|  | Systolic arterial pressure, mmHg | 122 [108, 132] | 122 [111, 129] | 0.916 |
|  | Diastolic arterial pressure, mmHg | 77 [68, 83] | 71. [66, 79] | 0.117 |
|  | Heart rate, bpm | 79 [71, 86] | 83 [71, 91] | 0.576 |
|  | Physiological variables at the end of SBT | | | |
|  | PEEP, cmH_2_O | 0 [0, 0] | 0 [0, 0] | 1.000 |
|  | PS, cmH_2_O | 7 [7, 7] | 7 [7, 7] | 1.000 |
|  | VT/PBW | 8.2 [7.5-8.6] | 7.5 [7.1-8.2] | 0.060 |
|  | FiO2 | 0.35 [0.30, 0.40] | 0.35 [0.30, 0.40] | 0.730 |
|  | Respiratory rate, bpm | 21 [19-23] | 23 [20-24] | 0.815 |
|  | RSBI | 49 [42-58] | 53 [42-61] | 0.529 |
|  | P0.1 | 2.7 [2.5-3.1] | 2.9 [2.5-3.1] | 0.024 |
|  | Pocc | 10 [9-13.8] | 14 [12-18] | 0.011 |
|  | Arterial pH | 7.42 [7.40-7.44] | 7.44 [7.43-7.45] | 0.700 |
|  | PaO2, mm Hg | 101 [98-105] | 94 [91-104] | 0.182 |
|  | PaCO2, mm Hg | 43 [41-44] | 41 [40.2-43.8] | 0.958 |
|  | Systolic arterial pressure, mmHg | 127 [120-134] | 125 [111-130] | 0.377 |
|  | Diastolic arterial pressure, mmHg | 78 [74.2-83] | 75 [73-79] | 0.156 |
|  | Heart rate, bpm | 78 [77-82] | 78.5 [75-83] | 0.477 |
|  |  |  |  |  |

Results are expressed as median [IQR] for continuous variables and number (percentage) for categorical variables. Abbreviations: APACHE II, Acute Physiology and Chronic Health Evaluation II; ICU, intensive care unit; MV, mechanical ventilation; RASS, Richmond Agitation and Sedation Scale; PBW, predicted body weight; PEEP, positive-end expiratory pressure; PS, pressure support delivered by ventilator; V_T_, tidal volume; FiO_2_, inspiratory fraction of oxygen; PaO_2_, arterial oxygen partial pressure; PaCO_2_, arterial carbon dioxide partial pressure. P0.1: airway occlusion pressure at 100 ms. Pocc, occlusion pressure measured during a sudden end-expiratory occlusion.^a^ APACHE II score was calculated from 17 variables. Scores range from 0 to 71 points, with higher scores indicating more severe disease. ^b^Calculated as weight in kilograms divided by height in meters squared. ^c^ Calculated as: 50 [or 45.5 for women] +0.91×[Height[cm]−152.4].

**Table E6. Univariate logistic regression for risk of re-intubation.**

|  | Oro-nasal NIV | | Full-Face NIV | | |
| --- | --- | --- | --- | --- | --- |
|  | **OR (95%CI)** | **P value** | | **OR (95%CI)** | **P value** |
| *Demographics* |  |  | |  |  |
| Sex (Male) | 0.51 (0.15–1.76) | 0.286 | | 0.51 (0.15–1.76) | 0.286 |
| Age | 0.98 (0.91–1.05) | 0.570 | | 0.98 (0.91–1.05) | 0.570 |
| Weight (kg) | 0.99 (0.96–1.02) | 0.586 | | 0.99 (0.96–1.02) | 0.586 |
| Predicted body weight, kg | 0.99 (0.94–1.04) | 0.676 | | 0.99 (0.94–1.04) | 0.676 |
| Body Mass Index | 1.00 (0.86–1.17) | 0.983 | | 1.00 (0.86–1.17) | 0.983 |
| *Non-occluded breaths* |  |  | |  |  |
| PEEP | 1.02 (0.41–2.58) | 0.964 | | 1.19 (0.52–2.71) | 0.672 |
| Ppeak, | 1.12 (0.58 – 2.18) | 0.733 | | 1.28 (0.94–1.74) | 0.110 |
| V_T_ / PBW, | 0.95 (0.49 – 1.84) | 0.882 | | 1.09 (0.57 – 2.0) | 0.793 |
| Respiratory Rate, breaths/min | 1.04 (0.92–1.14) | 0.391 | | 0.94 (0.83–1.06) | 0.318 |
| Air Leak | 1.02 (0.53 – 1.96) | 0.951 | | 1.32 (0.84 – 2.4) | 0.652 |
| Actual ΔPes | 0.75 (0.62–0.89) | **0.001** | | 0.66 (0.60–0.88) | **0.001** |
| ΔPL,dyn, | 1.29 (1.09–1.53) | **0.003** | | 1.39 (1.15–1.69) | **<0.001** |
| Clung,dyn, | 0.86 (0.78–0.95) | **0.005** | | 0.88 (0.81–0.96) | **0.005** |
| *Occlusion-derived parameters* |  |  | |  |  |
| Pocc, | 0.78 (0.67–0.90) | **0.001** | | 0.71 (0.59–0.86) | **<0.001** |
| Pocc, pneumotach | 0.81 (0.71–0.93) | **0.001** | | 0.74 (0.61–0.89) | **<0.001** |
| P0.1, es | 1.20 (1.06–1.35) | **0.002** | | 0.78 (0.66–0.90) | **0.001** |
| P0.1, vent, | 2.15 (1.21–3.81) | 0.080 | | 2.01 (0.54–2.5) | 0.171 |
| P0.1, pneumotach, | 1.70 (1.36–2.15) | 0.012 | | 1.22 (0.66–2.41) | 0.037 |
| Pplat, vent, | 0.91 (0.72–2.02) | 0.522 | | 1.72 (1.21–3.53) | 0.664 |
| Pplat,0.3s | 0.71 (0.39–1.35) | 0.320 | | 1.30 (0.95–1.77) | 0.093 |
| Pplat,1s, | 0.87 (0.68–1.12) | 0.307 | | 1.32 (1.02–1.69) | 0.290 |
| *Predicted Values derived from occlusion* |  |  | |  |  |
| Predicted ΔPes | 0.71 (0.57–0.87) | **0.001** | | 0.66 (0.52–0.84) | **<0.001** |
| Predicted ΔPL,dyn | 1.34 (1.10–1.63) | **0.004** | | 1.52 (1.20–1.93) | **<0.001** |
| Predicted Clung, dyn | 0.87 (0.78–0.96) | **0.007** | | 0.86 (0.77–0.96) | **0.005** |

Abbreviation: NIV: noninvasive ventilation; Paw: airway pressure; PEEP: positive end-expiratory pressure; Ppeak: Peak airway opening pressure; V_T_/PBW: inspired tidal volume normalized to predicted body weight; Pes: esophageal pressure; Actual ΔPes: maximal swing in esophageal pressure recorded during non-occluded breath (reference metric for inspiratory effort); P0.1,es: Pes drop 100 ms after the onset of inspiratory effort (reference metric for respiratory drive);PL: transpulmonary pressure; ΔPL,dyn: Dynamic transpulmonary driving pressure; Clung,dyn: Dynamic lung compliance; Pocc: airway occlusion pressure; P0.1: airway occlusion pressure at 100 ms; Pplat: Inspiratory plateau pressure recorded at 0.3 s (Pplat,0.3s) and 1.0 s (Pplat,1s) from the start of the end-inspiratory hold. Predicted ΔPes = K x Pocc, where Pocc is airway occlusion pressure; Predicted ΔPL,dyn= [Pressure support – (K x Pocc)]; Predicted Clung,dyn = the ratio between Expiratory tidal volume and Predicted ΔPL,dyn.

**Figure E2:** Scatterplots (left) show the concordance between ventilator and pneumotach in measuring Pocc. Bland–Altman plots (right) display agreement between measurements; the solid horizontal line indicates the mean bias and dashed lines the limits of agreement.

**Figure E3:** Bland–Altman plots (right) display agreement between methods; the solid horizontal line indicates the mean bias and dashed lines the limits of agreement.

**Figure E4:** Scatterplots (left) show the concordance between ventilator and pneumotach in measuring Pplat. Bland–Altman plots (right) display agreement between measurements; the solid horizontal line indicates the mean bias and dashed lines the limits of agreement.

**Figure E5.** Distribution of Pplat patterns (stable, incremental, decremental); bars show the number of patients and percentages the proportion of stable and unstable Pplat and stable and unstable traces per group.

**Figure E6:** Scatterplots showing association between variables; the solid horizontal line indicates linear regression.

**Figure E7. Complementary analysis for respiratory drive.**

Association between ActualΔPes/dt and P0.1,es (top panel), ventilator-derived P0.1 (middle panel), and pneumotachograph-derived P0.1 (bottom panel) during oro-nasal and full-face NIV. P0.1,es and pneumotachograph-derived P0.1 were strongly associated with ΔPes/dt in both interfaces, whereas ventilator-derived P0.1 showed no meaningful association. Solid lines represent model-estimated relationships and shaded areas their 95% confidence intervals.

**OCCLUSION PRESSURE DURING NON-INVASIVE VENTILATION: BENCH TESTS**

**Methods:**

Two mechanical ventilators were tested: Evita V800 (Dräger, Lübeck, Germany) and Servo-I (Getinge, Göteborg, Sweden). The patient circuit was attached to a face-mask sealed on a manikin head with realistic airways (Kernel, Rouen, France). Face-mask was adjusted to target inspiratory leaks around 30%. This manikin head was connected to a test lung (ASL 5000, Ingmar Medical). The compliance and the resistance were set at 50 mL/cmH_2_O and 5 cmH_2_O/L/s, respectively. To simulate spontaneous breathing on the ASL 5000, three inspiratory efforts were tested with a maximum muscular pressure of 5, 10 and 20 cmH₂O.

Occlusion pressure (Pocc) values were measured in pressure support ventilation during an expiratory hold in invasive and non-invasive modes with the same ventilator settings as follows: positive end expiratory pressure (PEEP) of 5 or 10 cmH_2_O, pressure support of 8 cmH_2_O, expiratory trigger of 25%. A pressure sensor and a flow sensor were placed at the Y-piece of the patient circuit to record pressure and flow, respectively.

**Results:**

Table E7:

| **Ventilator** | **PEEP (cmH2O)** | **Maximum Muscular pressure (ASL) (cmH20)** | **Pocc**  **invasive mode (cmH2O)** | **Pocc**  **non invasive mode (cmH2O)** | **Absolute delta Pocc (cmH2O)** |
| --- | --- | --- | --- | --- | --- |
| Evita V800 | 5 | 5 | -4.6 | -4.7 | 0.1 |
|  | 10 | 5 | -5.25 | -5.7 | 0.45 |
|  | 5 | 10 | -7.9 | -7.9 | 0 |
|  | 10 | 10 | -9.4 | -9.45 | 0.05 |
|  | 5 | 20 | -14.35 | -14.4 | 0.05 |
|  | 10 | 20 | -15.5 | -15.2 | 0.3 |
| Servo-I | 5 | 5 | -4.3 | -4.65 | 0.35 |
|  | 10 | 5 | -5 | -5.4 | 0.4 |
|  | 5 | 10 | -7.25 | -7.8 | 0.55 |
|  | 10 | 10 | -8.65 | -9.3 | 0.65 |
|  | 5 | 20 | -13.3 | -13.55 | 0.25 |
|  | 10 | 20 | -14.35 | -15.1 | 0.75 |

**Figure E8: Pressure and flow tracings comparing invasive and non-invasive modes during occlusion pressure manoeuver**

**Figure E8*:*** This figure displays pressure and flow tracings according to time of a ventilator (Evita V800) in pressure support ventilation during an expiratory hold permitting to measure occlusion pressure. For each panel, tracings obtained with an invasive mode are displayed in blue while tracings obtained with a non-invasive mode are displayed in orange. The left panel corresponds to results with a PEEP of 5 cmH_2_O while the right panel shows results with a PEEP of 10 cmH_2_O.
